# Structural changes in starch and protein of brown, red, and black rice flours induced by steam-assisted heat treatment

**DOI:** 10.1016/j.fochx.2026.104688

**Published:** 2026-10-27

**Authors:** C. Pintilii, L. Mihaly Cozmuta, F. Popa, C. Bîrleanu, M. Pustan, M. Baia, K. Magyari, Z. Szakacs, C. Nicula, A. Peter, A. Mihaly Cozmuta

**Affiliations:** a Technical University of Cluj-Napoca, North University Center of Baia Mare, Victoriei Str. 76, Baia Mare, Romania; b Technical University of Cluj-Napoca, Department of Materials Science and Engineering, Blv. Muncii 103-105, 400641 Cluj-Napoca, Romania; c Technical University of Cluj-Napoca, MicroNano Systems Laboratory, Mechanical Systems Engineering Department, Blv. Muncii Nr. 103-105, EUT+ Institute of Nanomaterials & Nanotechnologies EUTINN, European University of Technology, European Union, 400641 Cluj-Napoca, Romania; d Faculty of Physics, Babes-Bolyai University, Mihail Kogalniceanu Str. 1, 400084 Cluj-Napoca, Romania; e Interdisciplinary Research Institute on Bio-Nano-Sciences, Babes-Bolyai University, Treboniu Laurian Str. 42, 400271 Cluj-Napoca, Romania

**Keywords:** Rice, Steam-assisted heat treatment, Starch crystallinity, Protein secondary structure, FTIR spectroscopy, XRD, AFM, SEM, Digestibility

## Abstract

This study investigated the effects of steam-assisted heat treatment on starch and protein structural changes in brown, red, and black rice flours. Starch molecular order and crystallinity were assessed using FTIR, XRD, AFM, SEM, image analysis, and digestibility assays, while protein structure and thiol-disulfide chemistry were evaluated by FTIR and chemical analyses. FTIR indicated increased starch short-range order, with crystallinity rising from ∼62% to ∼67% in brown and red rice starches and from 65.85% to 69.01% in black rice starch. XRD revealed greater increases in long-range crystallinity in brown (25.44% to 47.63%) and red (27.10% to 42.81%) rice starches, with a more moderate increase in black rice. These structural changes promoted resistant starch formation, most prominently in brown rice, followed by red and black rice. Protein analyses showed greater structural stability in red and black rice, whereas brown rice proteins exhibited greater unfolding and aggregation following treatment.

## Highlights

• Steam-assisted heating rapidly increases starch crystallinity in rice flours.
• Black rice starch shows higher native crystallinity and limited reorganization.
• Brown and red rice starches form amylose–lipid complexes after treatment.
• Heat treatment alters starch surface roughness and pore size distribution.
• Protein secondary structure and disulfide bonding change by rice flour type.

## Graphical abstract

**Figure:**
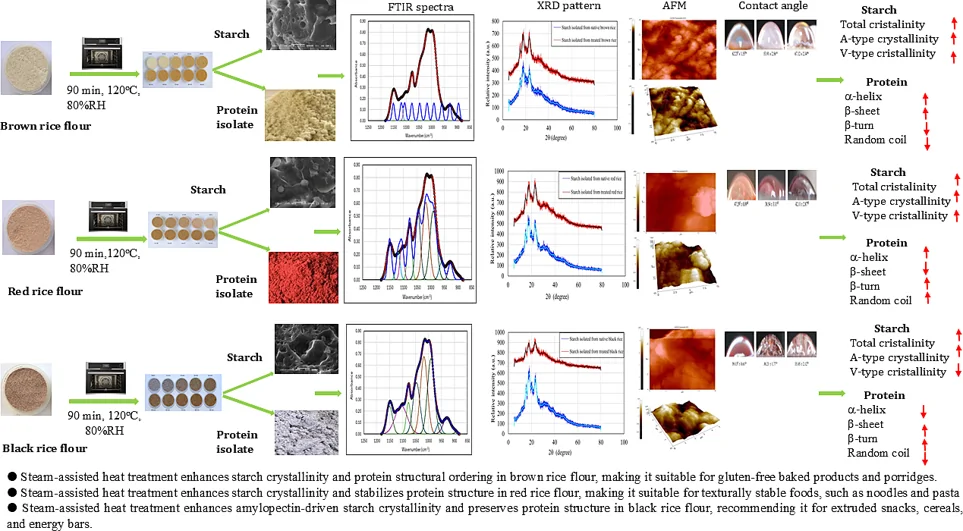

## 1 Introduction

Rice flour is widely used in food formulations due to its neutral flavor, gluten-free nature, and versatility in developing diverse textures. Its functional performance is strongly governed by the structural organization of starch and proteins, which can be modified through thermal processing. Depending on the processing temperature relative to starch gelatinization and moisture availability, thermal treatments are broadly classified into low-moisture, sub-gelatinization treatments and high-moisture, supra-gelatinization treatments, each inducing distinct molecular and supramolecular changes in rice flour components.

Low-moisture treatments, such as dry heat treatment, heat–moisture treatment, and annealing, modify the starch structure without complete gelatinization. Dry heat treatment (>100 °C, not intentional water or steam introduced) typically disrupts crystalline order, alters gelatinization behavior, and denatures proteins, leading to improved water absorption and textural properties in gluten-free baked products (Solaesa et al., 2022; Zhou et al., 2019). Heat–moisture treatment (HMT; 90–120 °C, 10–30% moisture) promotes reorganization of starch granules, enhances intermolecular interactions, and favors amylose-lipid complex formation, resulting in increased gelatinization temperatures, reduced swelling, and improved thermal and shear stability (Arns et al., 2015; Xiang et al., 2023; Van Hung et al., 2020; Thuengtung et al., 2021). Annealing, conducted at temperatures between the glass transition and gelatinization points under excess moisture, induces more subtle rearrangements of amylopectin double helices, narrowing gelatinization ranges and stabilizing starch structure, although its effects are generally less pronounced than those of HMT (Chung et al., 2009; Dias et al., 2010; Gomes et al., 2005; Lan et al., 2008; Waduge et al., 2006).

High-moisture treatments, including autoclaving, parboiling, and steaming, induce partial or complete starch gelatinization and extensive protein denaturation. Autoclaving (∼120 °C under pressure) disrupts crystalline regions, promotes starch retrogradation upon cooling, and enhances resistant starch formation, while also intensifying starch-protein interactions (Faridah et al., 2022; Li et al., 2019; Raungrusmee & Anal, 2019; Zheng et al., 2020). Parboiling, which combines soaking, steaming, and drying, partially gelatinizes starch, alters crystalline polymorphism, and produces a compact microstructure with reduced swelling and slower enzymatic hydrolysis (Gunaratne et al., 2013; Kumar et al., 2022; Tian et al., 2018).

Steam-assisted heat treatment (SAHT) can be considered an atmospheric-pressure moist-heat process in which rice flour is exposed to a humid environment generated by *in situ* water evaporation (Cao et al., 2024; Hu et al., 2024; Kim et al., 2021; Zhang et al., 2024). Unlike conventional heat–moisture treatment (HMT), which generally involves adjustment of the sample to a predetermined moisture level before heating (Arns et al., 2015; Xiang et al., 2023; Van Hung et al., 2020; Thuengtung et al., 2021), SAHT relies on moisture uptake from the surrounding humid atmosphere during treatment. It also differs from cooking and autoclaving, which involve substantially greater water availability and, in the case of autoclaving, elevated pressure (Faridah et al., 2022; Gunaratne et al., 2013). Accordingly, SAHT provides a specific moist-heat processing configuration in which thermal exposure and progressive moisture uptake occur simultaneously, while avoiding the extensive hydration and pressure conditions associated with conventional cooking or autoclaving. However, the structural response of whole rice flour to this treatment configuration, particularly across different structural levels of starch and protein, remains insufficiently characterized.

Similarly, limited information is available regarding its impact on protein secondary structure and thiol–disulfide chemistry. This knowledge gap is especially evident for pigmented rice flours such as red and black rice. These flours differ in their overall composition, including starch, protein, lipid, fiber, phenolic compounds, and mineral constituents (Table 1.SM). Comparative structural investigations of pigmented rice flours subjected to SAHT remain scarce. The brown, red, and black rice flours differ in their overall composition, including their starch, protein, lipid, fiber, and phenolic contents. Such compositional differences may contribute to variety-dependent responses to thermal and moisture exposure (Ozdal et al., 2013; Sangwongchai et al., 2021). However, the relative contributions of individual non-starch constituents to starch reorganization and protein structural changes remain insufficiently understood. We therefore hypothesized that SAHT would induce variety-dependent changes in starch crystallinity, protein structure, and *in vitro* starch digestibility. These responses were investigated using a multiscale analytical approach. The analytical techniques included FTIR spectroscopy, X-ray diffraction (XRD), atomic force microscopy (AFM), scanning electron microscopy (SEM), image analysis, and protein thiol–disulfide characterization. This combination allowed the evaluation of both short-range and long-range structural changes. By integrating starch crystalline features with protein secondary structure and redox chemistry, the study seeks to clarify variety-dependent structural responses under moist-heat conditions. The findings provide insight into variety-dependent structural responses of starch and proteins in pigmented rice flours. These results may contribute to the rational design of rice-based ingredients intended for thermally processed and gluten-free food applications.

**Table 1:** Results for the crystallinity degree of starch and secondary structure of protein in the investigated rice flours.

		Brown rice flour	Red rice flour	Black rice flour
Relative crystallinity degree resulted from FTIR spectra analysis, %				
Native state (Pintilii et al., 2025)		61.91 ± 0.33^bβ^	62.22 ± 0.47^bβ^	65.85 ± 0.51^aβ^
After steam-assisted heat exposure for 90 min		67.32 ± 0.45^bα^	66.98 ± 0.19^bα^	69.01 ± 0.76^aα^
Variation, %		+8.74	+7.65	+4.80
Crystallinity degree resulted from processing of XRD patterns, %				
		Total crystallinity degree, %		
Native state (Pintilii et al., 2025)		25.44 ± 0.51^bβ^	27.10 ± 0.77^bβ^	29.29 ± 0.10^aβ^
After steam-assisted heat exposure for 90 min		47.63 ± 1.96^aα^	42.81 ± 1.42^bα^	39.82 ± 1.59^bα^
Relative change in total crystallinity, %		+87.22	+57.96	+35.97
		A-type crystallinity, %		
Native state (Pintilii et al., 2025)		23.01 ± 0.92^bβ^	24.98 ± 0.89^abβ^	26.53 ± 0.72^aβ^
After steam-assisted heat exposure for 90 min		41.51 ± 1.49^aα^	38.28 ± 1.38^aα^	38.55 ± 1.20^aα^
Relative change in A-type crystallinity, %		+80.42	+53.21	+45.27
		*V*-type crystallinity, %		
Native state (Pintilii et al., 2025)		2.43 ± 0.11^bβ^	2.12 ± 0.05^cβ^	2.75 ± 0.1^aβ^
After steam-assisted heat exposure for 90 min		6.12 ± 0.25^aα^	4.53 ± 0.18^bα^	1.27 ± 0.05^cα^
Relative change in V-type crystallinity, %		+151.58	+113.99	−53.75
Secondary structure of protein resulted from FTIR spectra analysis, %				
Native state (Pintilii et al., 2025)	α-helix	22.45 ± 0.29^cβ^	40.56 ± 0.19^aβ^	26.00 ± 0.90^bα^
	β-sheet	37.54 ± 0.33^bβ^	34.33 ± 0.35^cα^	39.27 ± 1.21^aα^
	β-turn	22.42 ± 0.24^aα^	8.26 ± 0.25^cβ^	17.78 ± 0.40^bβ^
	random coil	17.59 ± 0.15^aα^	16.84 ± 0.35^bα^	16.96 ± 0.38^bα^
	α-helix and β-sheet, auxiliary (%)	59.99	74.89	64.27
After steam-assisted heat exposure for 90 min	α-helix	25.51 ± 0.45^bα^	45.00 ± 0.62^bα^	24.81 ± 0.62^bα^
	β-sheet	50.97 ± 0.45^aα^	28.52 ± 1.01^cβ^	39.95 ± 1.46^bα^
	β-turn	11.71 ± 0.45^bβ^	9.61 ± 0.43^cα^	22.90 ± 0.24^aα^
	random coil	11.82 ± 0.57^bβ^	16.87 ± 0.47^aα^	12.35 ± 0.62^bβ^
	α-helix and β-sheet, auxiliary (%)	76.48	73.52	64.76
Variation, %	α-helix	+13.63	+10.95	−4.58
	β-sheet	+35.78	−16.92	+1.73
	β-turn	−47.77	+16.34	+28.80
	random coil	−32.80	+0.17	−44.87
	α-helix + β-sheet, auxiliary	+27.49	−1.83	+0.76

Data are presented as mean ± standard deviation (*n* = 3). Values with different letters a, b and c in the same line for the same treatment of rice flours are significantly different (*p* < 0.05). Values with different letters α and β for the same rice flour and protein structure type but subjected to different exposure time are significantly different (*p* < 0.05). The α-helix + β-sheet value is reported as an auxiliary descriptive parameter and does not represent a single structural class. Interpretation of protein conformational changes is based primarily on the individual secondary-structure components.

## 2 Materials and methods

### 2.1 Chemicals and reagents

All chemicals utilized in this study were of analytical grade. The following reagents were sourced from Merck (Germany): sodium hydroxide, nitric acid, n-hexane, hydrochloric acid, Ellman's reagent (5,5′-dithio-bis-(2-nitrobenzoic acid)-DTNB), TRIS, sodium dodecyl sulfate, ethylenediaminetetraacetic acid (EDTA), glycine, cysteine, β-mercaptoethanol, urea, and trichloroacetic acid. Porcine pancreatin (protease activity: 350 FIP-U/g; lipase activity: 6000 FIP-U/g; amylase activity: 7500 FIP-U/g) and amyloglucosidase (4210 units) were purchased from Merck KGaA (Darmstadt, Germany) and ProtectVit (Romania), respectively.

### 2.2 Pigmented rice flours

Whole brown rice (SC Bio Navis), black rice (SC DECO Italy, from Thailand), and red rice (Biocultivars) were obtained from local markets, with their proximate compositions reported in Table 1.SM. These varieties were selected because they represent brown, red, and black rice types with reported differences in pigmentation and non-starch composition. Grains were inspected for broken kernels and impurities, milled using a Biovita grinder (Biovita ME-1, Romania), and sieved to <250 μm. Given that the moisture content of red and black rice flours did not differ significantly, all flours were standardized to 12%. To achieve this, distilled water was added only to the brown rice flour to adjust its moisture level from 10.78% to 12%, followed by homogenization and equilibration for 60 min in sealed containers at 25 °C.

### 2.3 Steam-assisted heat treatment (SAHT)

Rice flour samples (100 g) were accurately weighed and evenly distributed in a shallow pan to form a uniform layer of approximately 1 cm thickness. The samples were subjected to steam-assisted heat treatment (SAHT) in a conventional ventilated oven (Electrolux EOF6P76X2, Sweden; chamber volume 65 L) at 100 °C for 90 min under atmospheric pressure. To establish a moisture-enriched heating environment, 250 mL of distilled water was placed in a separate container inside the oven chamber before the treatment, allowing water to evaporate during heating. The rice flour was therefore exposed to heated, humid air generated by *in situ* water evaporation throughout the treatment.Relative humidity (RH) within the oven chamber was not directly measured due to instrumental limitations at elevated temperature. Consequently, the atmospheric conditions during SAHT cannot be defined in terms of a controlled or constant RH, and the treatment should be considered a standardized moist-heat exposure under the specific experimental configuration used in this study, rather than a fully controlled saturated-steam treatment. In the present study, SAHT refers specifically to a moist-heat treatment generated by *in situ* water evaporation in a ventilated oven. To ensure consistency among experiments, the same oven model and chamber volume, water volume (250 mL), treatment temperature (100 °C), treatment duration (90 min), sample mass (100 g), sample-layer thickness (∼1 cm), pan configuration, and sample position within the oven were used for all rice varieties. These parameters standardized the treatment setup, although the absence of direct RH monitoring means that the temporal and spatial distribution of humidity within the chamber cannot be confirmed.

Flour moisture content was determined before and after treatment by oven drying at 105 °C to constant weight according to AOAC 925.10. The initial moisture content of all flour samples was standardized to approximately 12%. Following 90 min of SAHT, moisture contents were 15.02 ± 0.15% for brown rice flour, 14.87 ± 0.33% for red rice flour, and 14.61 ± 0.12% for black rice flour. These measurements were used to characterize the net moisture change associated with the treatment but were not considered a substitute for continuous measurement of chamber RH. Additional observations regarding moisture evolution and visual changes during treatment are provided in Section 1.2.SM.

### 2.4 FTIR spectra

FTIR absorption spectra of the rice flours were recorded using a Perkin Elmer BX2 spectrometer (USA) equipped with an ATR Pike Miracle diamond crystal. The measurements were performed in the wavenumber range of 600 to 4000 cm^−1^ with a resolution of 4 cm^−1^, accumulating 50 scans. The spectral analysis focused on changes in the ordered structure of starch and the secondary structure of protein, specifically within the amide I band, in the ranges 900–1200 cm^−1^ and 1550–1700 cm^−1^, respectively. The ratio of the absorption bands at 1047 and 1020 cm^−1^ was used as an indicator of the relative short-range molecular order of starch. The FTIR-derived relative crystallinity degree (RCD) reflects local ordered structures and double-helical organization rather than long-range crystallinity measured by XRD. Prior to analysis, the FTIR spectra were normalized.

To quantitatively assess the starch and protein structures, spectral deconvolution was performed using Gaussian functions, with band positions determined through second derivative peak analysis (Pintilii et al., 2025). The iterative spectral deconvolution process for starch and protein secondary structures is illustrated in Fig. 2.SM. The individual components of the deconvoluted spectra were used to calculate the relative crystallinity degree (RCD) of starch (1047 cm^−1^ and 995 cm^−1^ corresponding to the crystalline form, and 1020 cm^−1^ for the amorphous form) (Wang et al., 2020). For protein, the ratio of secondary structure features such as β-sheet (extended ordered structure, 1636–1615 cm^−1^ for high frequency, 1700–1682 cm^−1^ for low frequency), random coil (disordered structure, 1645–1637 cm^−1^), α-helix (dense ordered structure, 1663–1646 cm^−1^), and β-turn (extended ordered structure, 1681–1664 cm^−1^) was determined (Zhao et al., 2021). To validate the results obtained from the FTIR spectral analysis, starch and protein were extracted from rice flours and subsequently characterized.

### 2.5 Starch isolation and characterization

Starch was extracted from rice flour using the alkaline method (Kraithong et al., 2018). Flour was treated with 0.05 M NaOH for 5 h at room temperature to remove proteins and lipids, followed by centrifugation at 3000 x*g* for 20 min. The sediment was washed, dried at 25 °C for 48 h, and equilibrated to 20% moisture at 25 °C for 3 h. The isolated starch was analyzed by XRD, scanning electron SEM, AFM and digestibility.

#### 2.5.1 X-ray diffraction (XRD)

X-ray diffraction (XRD) analysis was performed using a Shimadzu XRD-6000 (Kyoto, Japan) equipped with Cu-Kα radiation (λ = 1.54 Å) and a Ni filter to assess the starch crystallinity. The XRD patterns were collected in reflection mode (2θ) within a range of 5° to 80° at room temperature, with a scanning speed of 2°/min.

#### 2.5.2 Scanning electron microscopy (SEM)

The samples were initially mounted on a double-sided carbon adhesive tape, followed by the deposition of a gold layer to enhance their electrical conductivity. Gold sputtering was performed using a JEOL SmartCoater. Subsequent analysis was conducted using the topographic contrast provided by secondary electron signals in a scanning electron microscope (SEM). The imaging was carried out on a JEOL JSM 5600LV SEM at 15 kV, high vacuum, and secondary electron imaging (SEI) mode. The scale bar was used for spatial calibration, and the maximum Feret diameter of clearly distinguishable individual granules was measured using ImageJ (Schneider et al., 2012). Overlapping, fused, and partially visible granules at the image boundaries were excluded from the analysis. At least 30–50 granules per sample were analyzed, and the results were expressed as mean ± standard deviation.

#### 2.5.3 Image analysis

The distribution of surface voids and damaged regions was determined from SEM micrographs using ImageJ (Schneider et al., 2012). Images were calibrated using the SEM scale bar and converted to binary masks using Otsu thresholding. Dark regions corresponding to surface depressions, fissures, cracks, and collapsed areas generated during processing were quantified according to their projected area. Distributions were obtained from at least three micrographs per sample. To ensure comparability among starch types, fixed-size intervals of 0.757 μm were used for area classification.

#### 2.5.4 Atomic force microscopy (AFM)

Atomic Force Microscopy (AFM) measurements were performed using a Park Systems XE-70 atomic force microscope (Park Systems Corporation, Suwon, South Korea), in accordance with the methodology previously reported by Pustan et al. (2022). The instrument is equipped with a decoupled scanner architecture, which minimizes cross-talk between the scanning axes and ensures accurate height measurements during high-resolution surface imaging.

AFM topographic imaging was conducted in true non-contact dynamic mode (attractive regime) in order to avoid any mechanical interaction between the AFM tip and the starch surface. Surface characterization was carried out using PPP-NCHR silicon cantilevers (thickness 4 μm, width 30 μm, length 125 μm, nominal spring constant 42 N/m, nominal resonance frequency 330 kHz), which provide high operational stability and fast scanning capability. The free oscillation amplitude was set to 20 nm, and the amplitude set-point was adjusted to 2 nm to maintain stable non-contact conditions. Surface scans were acquired at a scan rate of 0.10 Hz. Topographic images were recorded over scan areas of 10 × 10 μm^2^. Prior to quantitative analysis, height data were processed by plane subtraction and line-by-line leveling to remove background tilt and scanning artifacts. Surface roughness was quantified using the arithmetical mean roughness (Ra) and the root mean square roughness (Rq), calculated from the leveled height maps. All AFM measurements were performed under controlled environmental conditions, at a temperature of 20 °C and a relative humidity of 40%, in a class 1000 cleanroom. An active anti-vibration stage was used to minimize external mechanical noise. For each sample, at least three to five independent surface regions were analyzed, and the reported roughness values represent the average of these measurements. AFM data processing and analysis were carried out using Park Systems XEP software. It should be noted that AFM is a surface-sensitive technique and that the obtained roughness parameters reflect nanoscale surface morphology rather than bulk structural properties.

#### 2.5.5 Digestion of starch

*In vitro* starch digestibility was determined using the method proposed by Chi, Li, Zhang, Chen and Li (2018) adapted to this study. Working solution A was prepared by dispersing 12 g of porcine pancreatin in 80 mL of deionized water, followed by centrifugation at 3000 ×*g* for 15 min. Working solution B was obtained by combining 3.15 mL of amyloglucosidase with 3.85 mL of deionized water. Subsequently, the fresh enzyme working solution was prepared by mixing 54 mL of solution A with 6 mL of solution B. One gram of starch (considered the total starch TS, dry basis), isolated from native rice flours and flours subjected to SAHT, was suspended in 20 mL of acetate buffer (pH 5.2). Subsequently, 5 mL of the enzyme working solution was added, and the mixture was incubated at 37 °C in a water bath under continuous stirring at 190 rpm. After 20 and 120 min of enzymatic hydrolysis, the enzyme was inactivated by the addition of 20 mL of 66% ethanol. The samples were then centrifuged at 4000 ×*g* for 5 min. The concentration of released glucose in the supernatant, was quantified using a glucose oxidase/peroxidase assay (BioSystems, 2026). Starch fractions, rapidly digestible starch (RDS), slowly digestible starch (SDS), and resistant starch (RS), were subsequently calculated using the following equations:

(1) RDS=G20×0.9

(2) $$\mathrm{SDS}=\left(G120-G20\right)\times 0.9$$

(3) RS=TS−RDS–SDS

### 2.6 Protein isolation and characterization

Protein was extracted from rice flour following Zhao et al. (2021). Flour was defatted with n-hexane (1:5 *w*/*v*) and sieved through a 60-mesh (0.250 mm) sieve. The sample was then suspended in 0.1 mol/L NaOH (1:9 *w*/*v*) and stirred at 25 °C for 4 h. After centrifugation at 6000 ×*g* for 30 min at 4 °C, the supernatant pH was adjusted to 5.5 with 2 mol/L HCl to precipitate proteins. The precipitate was collected by centrifugation (3000 ×*g*, 30 min), washed, neutralized, and dried at 30 °C for 24 h. The protein isolate was equilibrated to 20% moisture at 25 °C for 3 h prior to analysis of surface hydrophobicity and free sulfhydryl and disulfide bond contents.

#### 2.6.1 Quantification of free sulfhydryl content

Free sulfhydryl (-SH) content of rice protein isolate was measured according to Pareyt et al. (2010) with modifications. A 100 mg sample was mixed with 8 mL of buffer (2.5% sodium dodecyl sulfate, 4.0 mM EDTA, 92 mM glycine, and 86 mM Tris-HCl at pH 8.0) and incubated for 1 h. Then, 8 mL of 4 mg/mL Ellman's reagent was added, and the mixture was incubated at 25 °C in the dark for 30 min. After centrifugation at 10,000 ×*g* for 10 min, the absorbance of the supernatant was measured at 412 nm (UV/VIS Lambda 35, PerkinElmer, USA). Control samples without Ellman's reagent were used to correct for colour interference. Free -SH content was calculated from a cysteine standard curve (R^2^ = 0.9966) and expressed as mM/g.

#### 2.6.2 Quantification of disulfide bond content

Disulfide bond (SS) content was measured following Liu et al. (2023). A 300 mg sample of rice protein isolate was dispersed in 12 mL of pH 8.0 Tris-Gly-8 M Urea buffer, mixed for 1 h at room temperature, and centrifuged at 7500 rpm for 10 min at 4 °C. A volume of 3 mL of the supernatant was reacted with 0.3 mL of 50 mM β-mercaptoethanol in the dark for 1 h and precipitated with 12 mL cold acetone. The precipitate was washed three times with cold acetone to remove β-mercaptoethanol, dissolved in 10 mL Tris-Gly-8 M Urea, and mixed with 10 mL of 4 mg/mL Ellman's reagent. After 30 min in the dark, the mixture was centrifuged at 6000 ×*g* for 5 min, and the absorbance of the supernatant was measured at 412 nm (UV/VIS Lambda 35, PerkinElmer, USA). Control samples without Ellman's reagent were used to correct for background absorbance. Total -SH content was calculated from a cysteine standard curve (R^2^ = 0.9990), and disulfide bond content was determined using Eq. 4.

(4) $$\mathrm{SS}\;\left(\mathrm{mM}/g\right)=\left(\text{total}-\mathrm{SH}–\text{free}-\mathrm{SH}\right)/2$$

where total-SH is the total sulfhydryl content and free-SH is the free sulfhydryl content.

### 2.7 Statistical analysis

Experiments were performed in triplicate, and results are expressed as mean ± standard deviation. Statistical differences were evaluated by one-way ANOVA with Tukey's *post hoc* test (*p* < 0.05).

Principal component analysis (PCA) was used to assess differences among pigmented rice flours in starch relative crystallinity and protein secondary structure in rice samples (Statistica 7.0, StatSoft, USA). PCA reduced the dataset by transforming correlated variables into independent components, with PC1 capturing the majority of variation and subsequent components (*e.g.*, PC2) explaining remaining variance.

Because PCA was performed separately on only three rice varieties (brown, red, and black rice) within each experimental condition (native and SAHT-treated), the number of observations was limited. Consequently, the proportion of variance explained by the first two principal components is expected to be inherently high due to the restricted dimensionality of the dataset. Therefore, the percentage of variance explained by PC1 and PC2 should be interpreted primarily as a mathematical consequence of the small sample number rather than as independent evidence of particularly strong associations among the analyzed variables. The PCA results are thus intended as an exploratory visualization of sample relationships and variable patterns.

## 3 Results and discussion

### 3.1 Starch characterization

#### 3.1.1 FTIR spectra

Fig. 1 shows the evolution of relative crystallinity degree (RCD) in native starch isolated from brown, red, and black rice flours subjected to SAHT, and Table 1 presents ANOVA results for RCD values before and after treatment. The treatment caused a pronounced early increase in RCD for all flours, followed by a plateau. In brown and red rice starches, RCD rose rapidly from ∼62% at 0 min to 66–67% within the first 10–20 min, indicating rapid reorganization of local double-helical structures and increased short-range molecular order. After 20 min, RCD values exhibited only minor variations up to 90 min, suggesting that most of the increase in short-range molecular order occurred during the early stages of treatment.

**Fig. 1:**
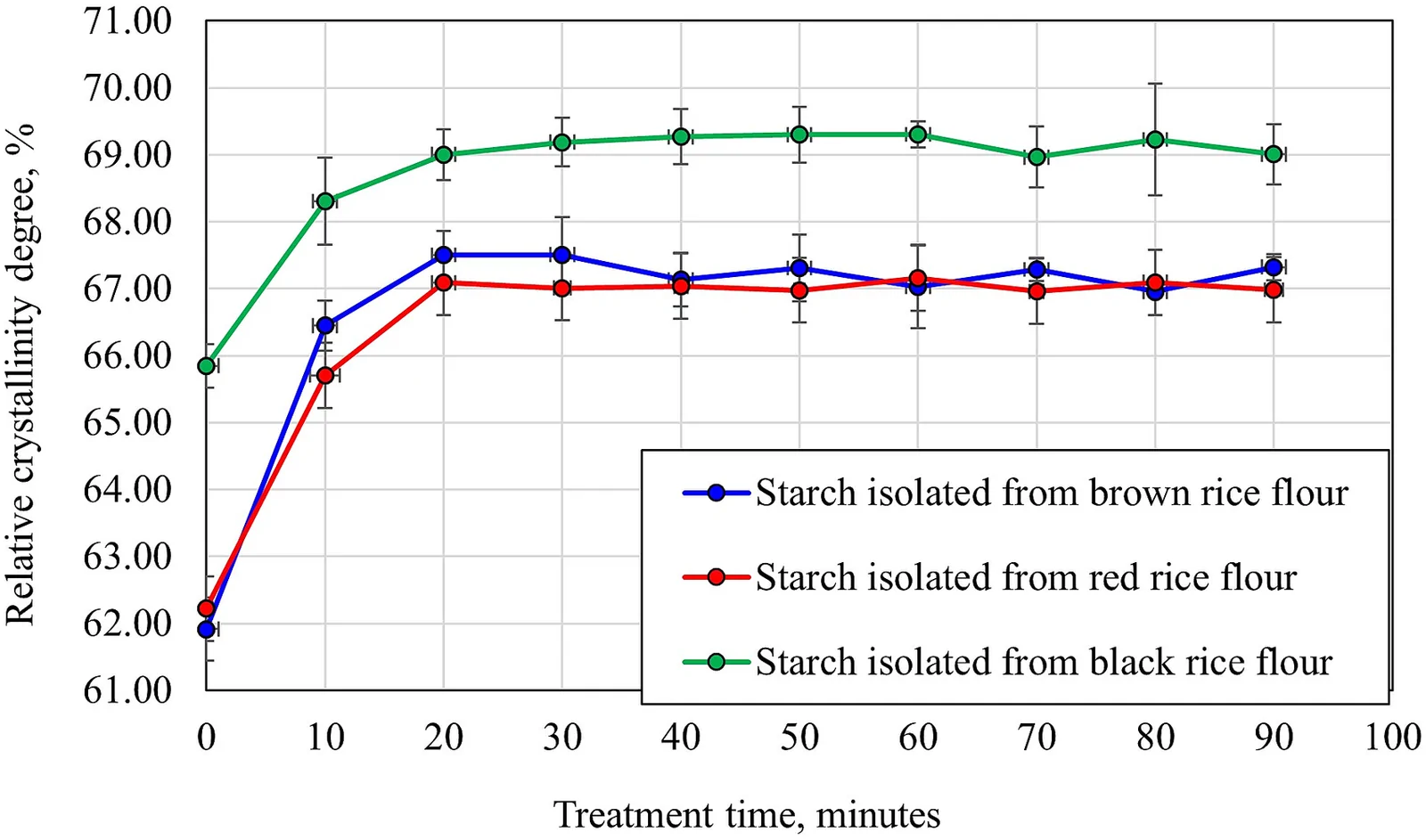
Changes in relative crystallinity degree of starch (RCD, %) in pigmented rice flours during steam-assisted heat treatment exposure:

Starch isolated from black rice flour exhibited higher RCD values than the other samples throughout SAHT, increasing from 65.85% initially to 69.00% within the first 20 min and showing only minor fluctuations at longer treatment times. This consistently higher crystallinity likely reflects greater intrinsic crystalline stability, possibly due to differences in molecular architecture or interactions with non-starch components characteristic of black rice. Minor fluctuations at extended times (≥ 70 min) were not significant. Overall, these results indicate that SAHT promotes the rapid development of short-range molecular order during the early stages of treatment, followed by structural stabilization. It should be noted that FTIR-derived RCD reflects local double-helical organization and should not be interpreted as a measure of long-range crystallinity, which was independently evaluated by XRD.

#### 3.1.2 XRD diffraction pattern and crystallinity

Fig. 2 shows the raw X-ray diffractograms of starch isolated from brown, red, and black rice flours before and after SAHT. All samples exhibited the typical A-type crystalline structure of cereal starches, characterized by strong diffraction peaks at ∼2θ 15° and 23°, and a less resolved double peak at 17–18°. These features are consistent with previous reports for rice starch (Bhat & Riar, 2019). A minor peak near 20° was also observed, indicative of *V*-type crystallinity associated with amylose–lipid complex formation (Sangwongchai et al., 2021). Total starch crystallinity, including the relative contributions of A-type and *V*-type phases, was quantified following Pintilii et al. (2025) and is summarized in Table 1. Unlike FTIR-derived RCD, which reflects short-range molecular organization, XRD quantifies long-range crystalline order arising from the packing of double helices into crystalline lattices. Therefore, changes observed by the two techniques are complementary rather than directly comparable.

**Fig. 2:**
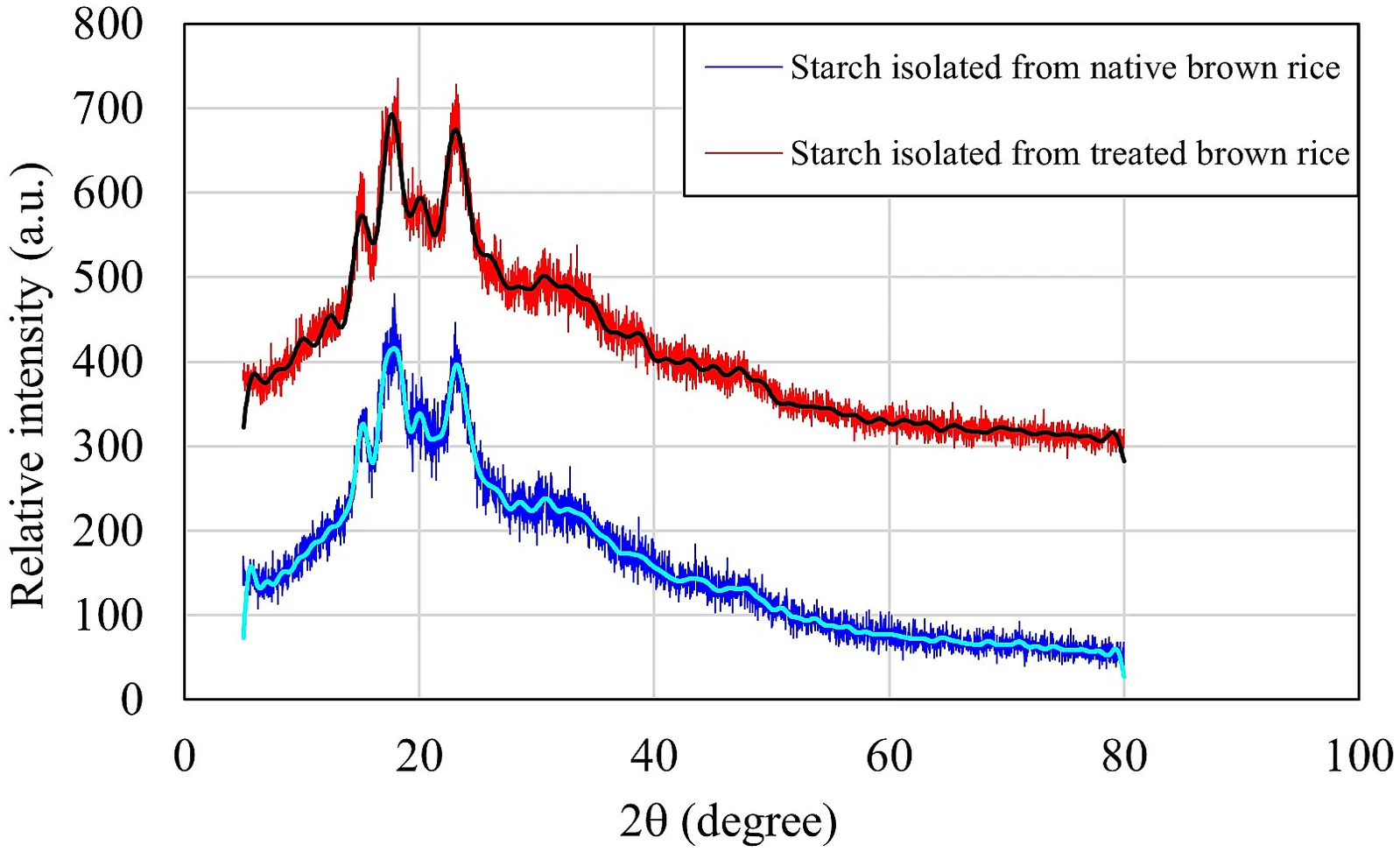

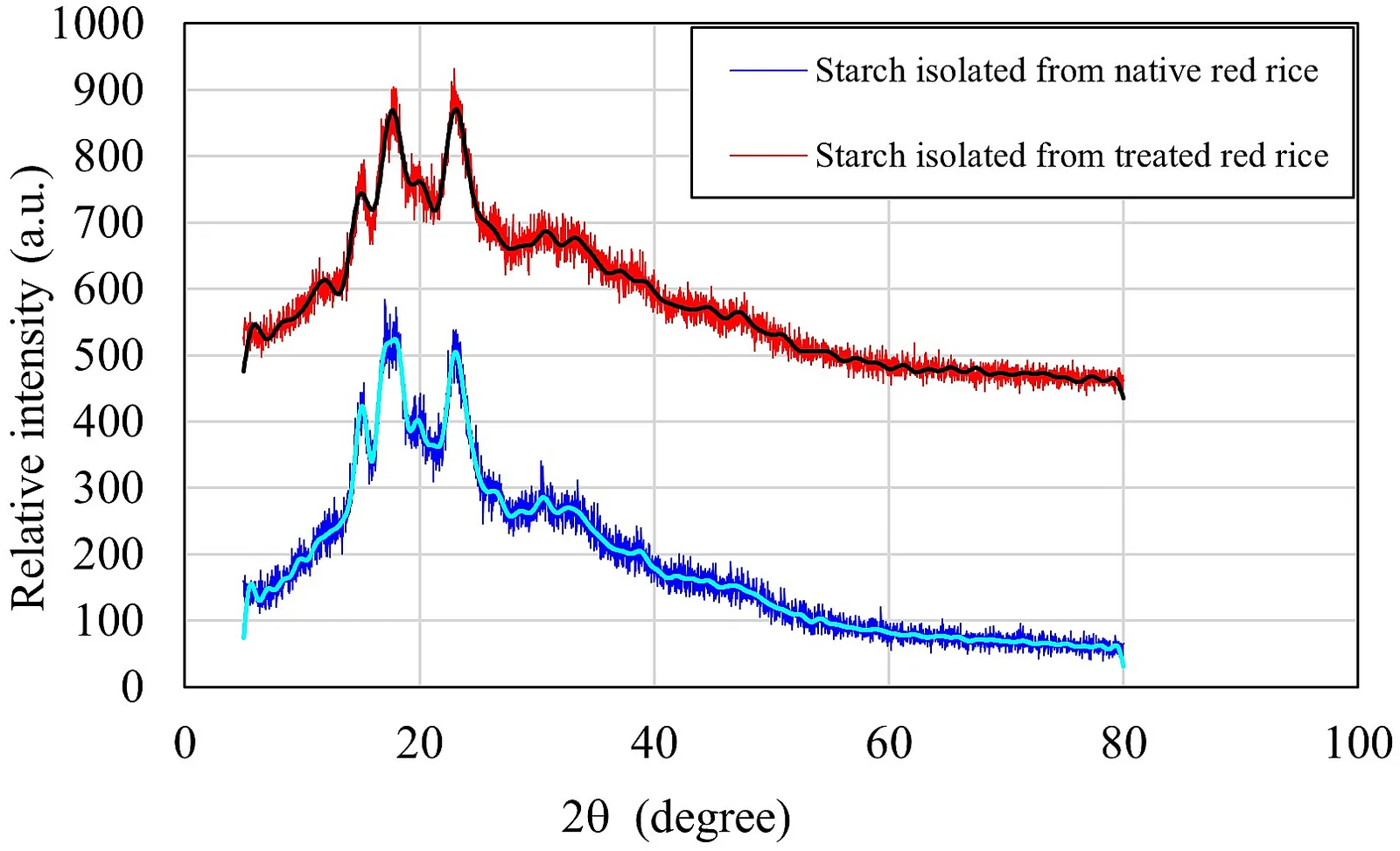

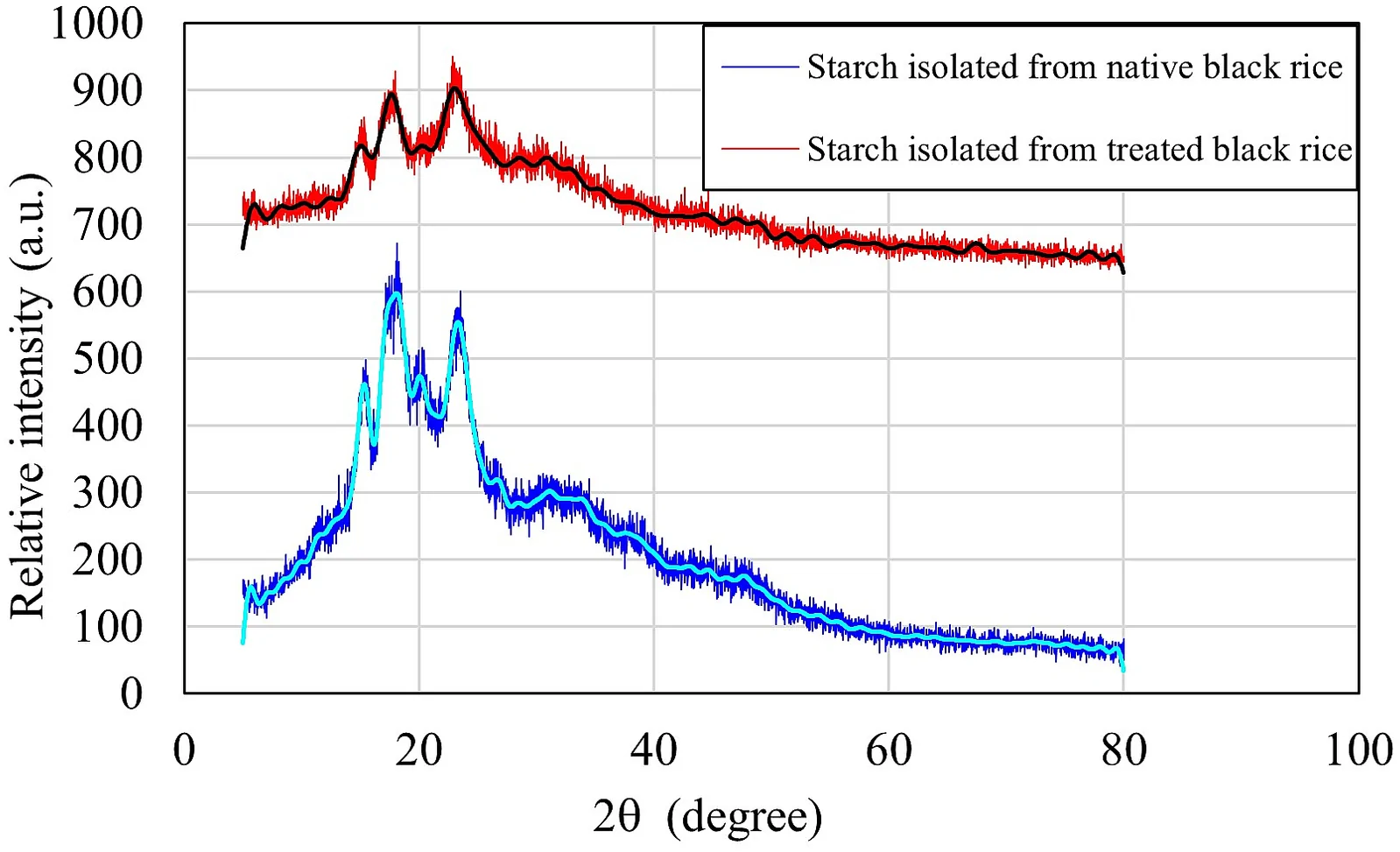
The XRD patterns of starches isolated from rice flours: a.Raw XRD patterns of brown rice flour before and after steam-assisted heat treatment exposure; b. Raw XRD patterns of red rice flour before and after steam-assisted heat treatment exposure; c. Raw XRD patterns of black rice flour before and after steam-assisted heat treatment exposure. (For interpretation of the references to colour in this figure legend, the reader is referred to the web version of this article.)

Analysis of XRD patterns showed that native black rice flour had the highest overall crystallinity (29.29%), exceeding that of brown (25.44%) and red rice flour (27.10%), which did not differ significantly. This trend is consistent with FTIR results. Crystallinity values in this study were lower than those reported by Bhat and Riar (2019), who found values of 34.53–38.55% across different rice varieties. This discrepancy may be related to methodological differences, including the approach used for crystallinity quantification (*e.g.*, peak deconvolution *versus* alternative diffraction profile analyses), differences in starch isolation procedures, or varietal characteristics of the rice samples.

Prolonged SAHT induced dual mechanisms of structural stabilization in brown and red rice starches. Native brown rice starch had moderate total crystallinity (25.44%), dominated by A-type regions (23.01%) with minor *V*-type contribution (2.43%). SAHT increased total crystallinity to 47.63% (+87.22% relative change), primarily through A-type crystallinity (+80.42% relative change), reflecting reorganization and perfection of amylopectin double helices. The increase in the *V*-type contribution (+151.58% relative change) is consistent with the possible formation or enhancement of amylose–lipid inclusion complexes. However, these complexes were not directly quantified in the present study. Native red rice starch showed intermediate total crystallinity (27.10%), mostly A-type (24.98%) with minor *V*-type (2.12%). After treatment, total crystallinity increased by 57.96%, mainly from A-type crystallinity (+53.21% relative change) and a moderate rise in *V*-type (+114% relative change). The smaller increase in crystallinity observed in red rice starch indicates a less pronounced change in long-range crystalline order than in brown rice starch. This difference may reflect the distinct composition and initial structural organization of the rice flours. However, the present data do not allow the individual contributions of water, lipid content, amylose availability, or other non-starch constituents to be distinguished. In both rice types, water likely acted as a plasticizer, increasing molecular mobility and facilitating structural rearrangements. Elevated temperature and prolonged exposure may have promoted partial disruption and subsequent reorganization of starch double helices, contributing to the observed increase in A-type crystallinity. These changes are consistent with mechanisms described by Arns et al. (2015) and Tang et al. (2019), in which hydrothermal treatment can increase double-helix mobility, promote transient disruption of hydrogen-bonding interactions, and facilitate subsequent reorganization into more ordered structures. The observed increase in crystallinity is consistent with the possibility that a larger fraction of amylopectin chains participated in ordered structures during treatment. As reported in the literature (Bienkiewicz & Kołakowska, 2004; Putseys et al., 2010), amylose–lipid inclusion complexes restrict amylose mobility and solubility, preventing double-helix formation and limiting retrogradation. One possible mechanistic explanation, which remains to be experimentally verified, is that the increase in A-type crystallinity reflects enhanced amylopectin reorganization and crystal perfection while amylose-driven retrogradation remains limited, particularly in the presence of amylose–lipid complex formation. The formation of these complexes also contributes to mechanical and thermal stabilization of the starch structure, consistent with reports that hydrothermal treatment strengthens amylose–amylopectin interactions (Hoover & Vasanthan, 1994). Preferential degradation or reorganization of amorphous regions under high moisture and temperature may initially reduce short-range order, but subsequent amylopectin recrystallization and amylose–lipid complex formation increase overall structural order. In brown and red rice starches, these two concurrent processes contribute to the development of a more ordered matrix with enhanced mechanical strength and thermal stability. The prolonged thermal exposure under the present moist-air conditions promoted both mechanisms compared with milder treatments, producing a pronounced increase in total crystallinity and structural stability in brown rice starch and a moderate, yet significant, enhancement in red rice starch. These mechanistic interpretations are inferred from the crystallinity changes observed by XRD and from mechanisms reported in previous hydrothermal starch studies. Because thermal transitions were not directly measured in the present work, techniques such as differential scanning calorimetry (DSC) would be required to verify the proposed processes of double-helix disruption, molecular reorganization, and recrystallization. In contrast to brown and red rice, black rice starch showed distinct crystallization behavior under SAHT. Native black rice starch had the highest total crystallinity (29.29%), dominated by A-type regions (26.53%) with a minor *V*-type fraction (2.75%). After prolonged treatment, total crystallinity increased to 39.82% (+35.97% relative change), primarily due to A-type crystallinity (26.53% → 38.55%), indicating ongoing amylopectin reorganization. The magnitude of this increase was lower than in brown and red rice, suggesting that black rice starch has a more ordered native structure that limits further recrystallization. The decrease in the *V*-type contribution (2.75% → 1.27%, −53.75% relative change) may reflect a reduction or structural alteration of pre-existing amylose–lipid complexes, although this interpretation requires confirmation by an independent measurement of amylose–lipid complex content.

The structural modifications induced by SAHT can be placed within the broader context of hydrothermal starch processing. Conventional heat–moisture treatment (HMT) has been reported to promote molecular reorganization, increase structural stability, and reduce starch digestibility while largely preserving the native granular and crystalline framework of rice starch. For example, Van Hung et al. (2020) observed that HMT-treated unpolished red rice starch retained its crystalline pattern while exhibiting reduced digestibility, whereas Arns et al. (2015) reported increased thermal and mechanical stability associated with starch structural reorganization. In contrast, parboiling generally involves greater water availability and partial starch gelatinization. Gunaratne et al. (2013) reported that parboiling increased susceptibility to enzymatic hydrolysis in several rice varieties and produced only limited changes in resistant starch content, indicating a structural response different from the increase in resistant starch observed after SAHT. Autoclaving-based hydrothermal treatments represent an even more intensive process because of the combined effects of high moisture and pressure. These treatments typically promote extensive gelatinization followed by retrogradation during cooling and are widely used to increase resistant starch formation (Faridah et al., 2022). Compared with these conventional hydrothermal treatments, SAHT appears to occupy an intermediate position, providing sufficient moisture to facilitate starch and protein reorganization and increase crystalline order, while avoiding the extensive gelatinization commonly associated with parboiling and autoclaving. This interpretation is further supported by the retention of the A-type crystalline pattern in all rice flours after treatment, despite the substantial increases in crystallinity and resistant starch observed in the present study.

From a processing perspective, the present SAHT should be distinguished from conventional HMT primarily by the mode of moisture delivery rather than by a fundamentally different molecular mechanism. In the present treatment, moisture was progressively absorbed from the humid oven environment, increasing from 12% initially to approximately 15% after 90 min, whereas conventional HMT generally involves adjustment of the sample to a predetermined moisture level before heating. The atmospheric-pressure conditions and limited water availability also distinguish SAHT from cooking and autoclaving, which provide substantially greater water availability and, in the latter case, elevated pressure. Under the present conditions, water likely acted as a plasticizer, increasing molecular mobility and facilitating partial disruption and subsequent reorganization of starch structures. The rapid increase in FTIR-derived short-range order during the first 10–20 min followed by only minor variations at longer treatment times suggests that the major structural reorganization occurred during the early stages of treatment. The plateau observed by FTIR indicates stabilization of local double-helical arrangements, whereas the larger changes detected by XRD reflect continued development of long-range crystalline organization during treatment. Thus, the observed changes are interpreted as the combined result of thermal exposure and progressive moisture uptake rather than as evidence of a mechanism unique to SAHT.

In black rice starch, the decrease in the *V*-type crystallinity contribution following SAHT may indicate changes in crystalline structures associated with amylose–lipid interactions. However, *V*-type crystallinity alone does not establish the formation, disruption, or stability of amylose–lipid complexes. The increase in A-type crystallinity indicates that the overall structural response was dominated by changes in A-type crystalline organization. Differences in the composition and initial molecular organization of the rice flours may have contributed to the variety-dependent responses observed during treatment. Nevertheless, the present data do not establish the specific contribution of phenolic compounds or other non-starch constituents to these changes. Independent quantification of amylose–lipid complexes and targeted assessment of phenolic interactions would be required to test these proposed mechanisms.

Unlike brown and red rice, the reduction in *V*-type crystallinity indicates a single dominant mechanism driven by amylopectin reorganization, with minimal contribution from amylose–lipid complexes. This behavior reflects the influence of native molecular organization, starch composition, and non-starch components on hydrothermal response. Overall, black rice starch shows a moderate increase in total crystallinity and improved structural stability. The retention of A-type polymorph after treatment aligns with previous reports, confirming its structural stability under SAHT conditions (Khunae et al., 2007). Studies on isolated rice starch often report a reduction or stabilization of A-type crystallinity due to partial disruption of amylopectin double helices (Khunae et al., 2007). In the present study, A-type crystallinity increased in brown, red, and black rice flours after SAHT (+45.27–80.42% relative change). This difference likely reflects the use of whole rice flours, where starch–protein–lipid interactions and matrix-induced constraints promote molecular reorganization and crystal perfection. Such interactions enhance A-type crystalline order during treatment, leading to an apparent increase rather than the reduction observed in isolated starch systems.

#### 3.1.3 Atomic force microscopy (AFM)

Fig. 3 presents representative 2D and 3D AFM topographic images of starch isolated from native and SAHT-treated brown and black rice flours, together with the corresponding surface roughness parameters (Ra and Rq). The complete AFM dataset is provided in Fig. 4.SM. The AFM analysis reveals pronounced differences in surface morphology among rice types in the native state, as well as distinct, rice-dependent surface responses to hydrothermal processing.

**Fig. 3:**
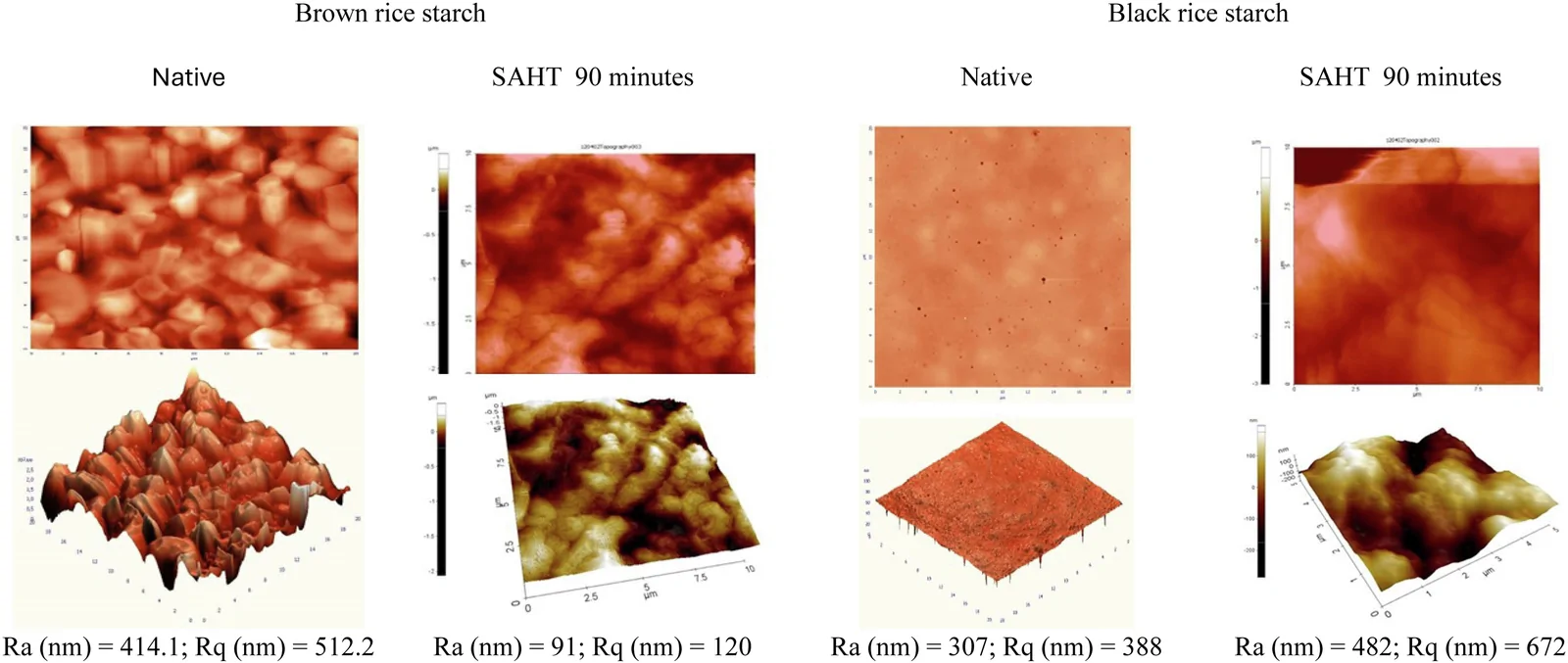
Representative 2D and 3D AFM topographic images of starch granules isolated from brown and black rice flours before and after steam-assisted heat treatment (SAHT, 100 °C, 90 min). Surface roughness parameters (Ra and Rq) are indicated for each sample. Brown rice starch exhibited pronounced surface smoothing after treatment, whereas black rice starch showed increased nanoscale roughness. The complete AFM dataset, including images of red rice starch, is provided in Fig. 4.SM. (For interpretation of the references to colour in this figure legend, the reader is referred to the web version of this article.)

**Fig. 4:**
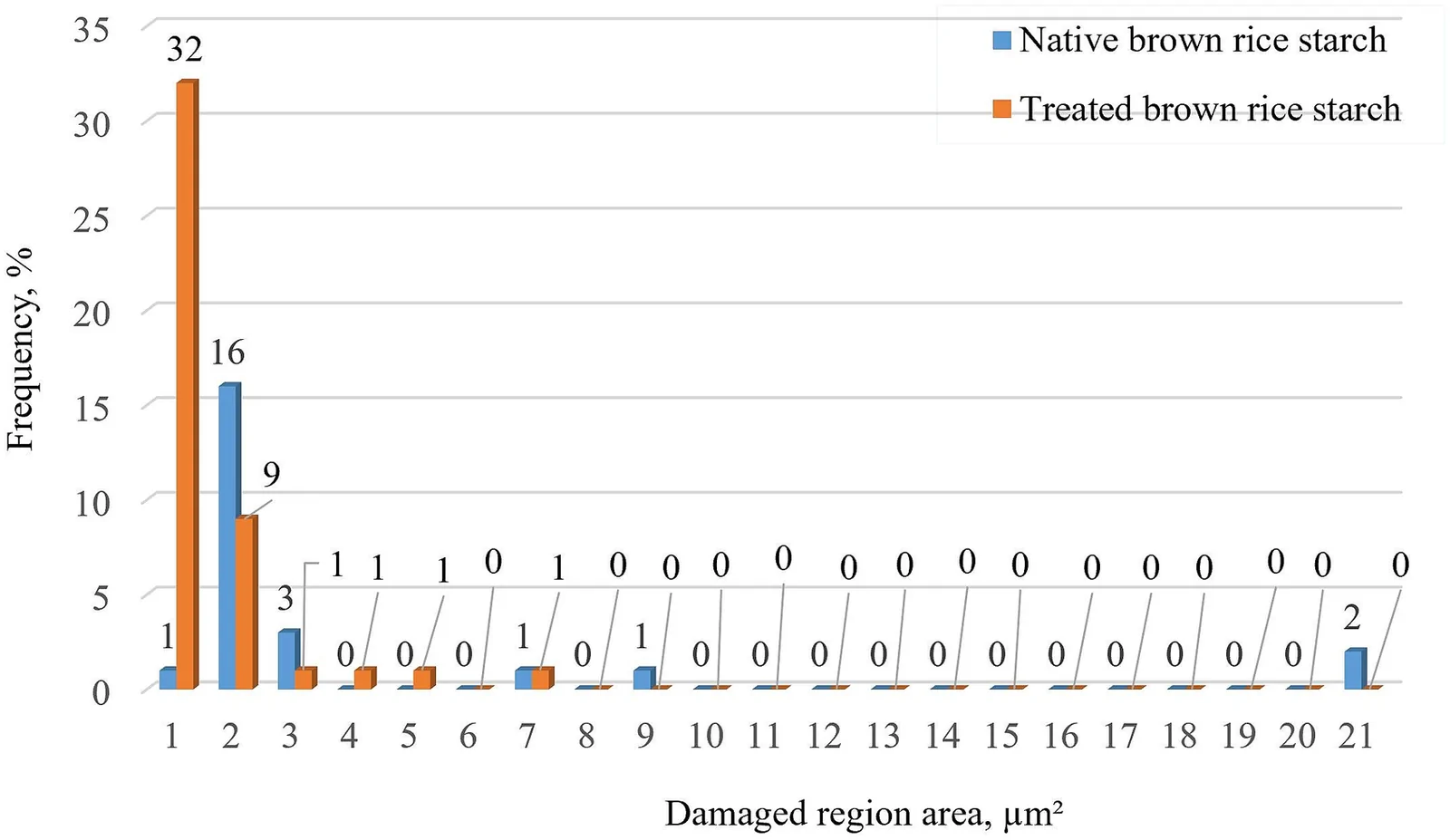

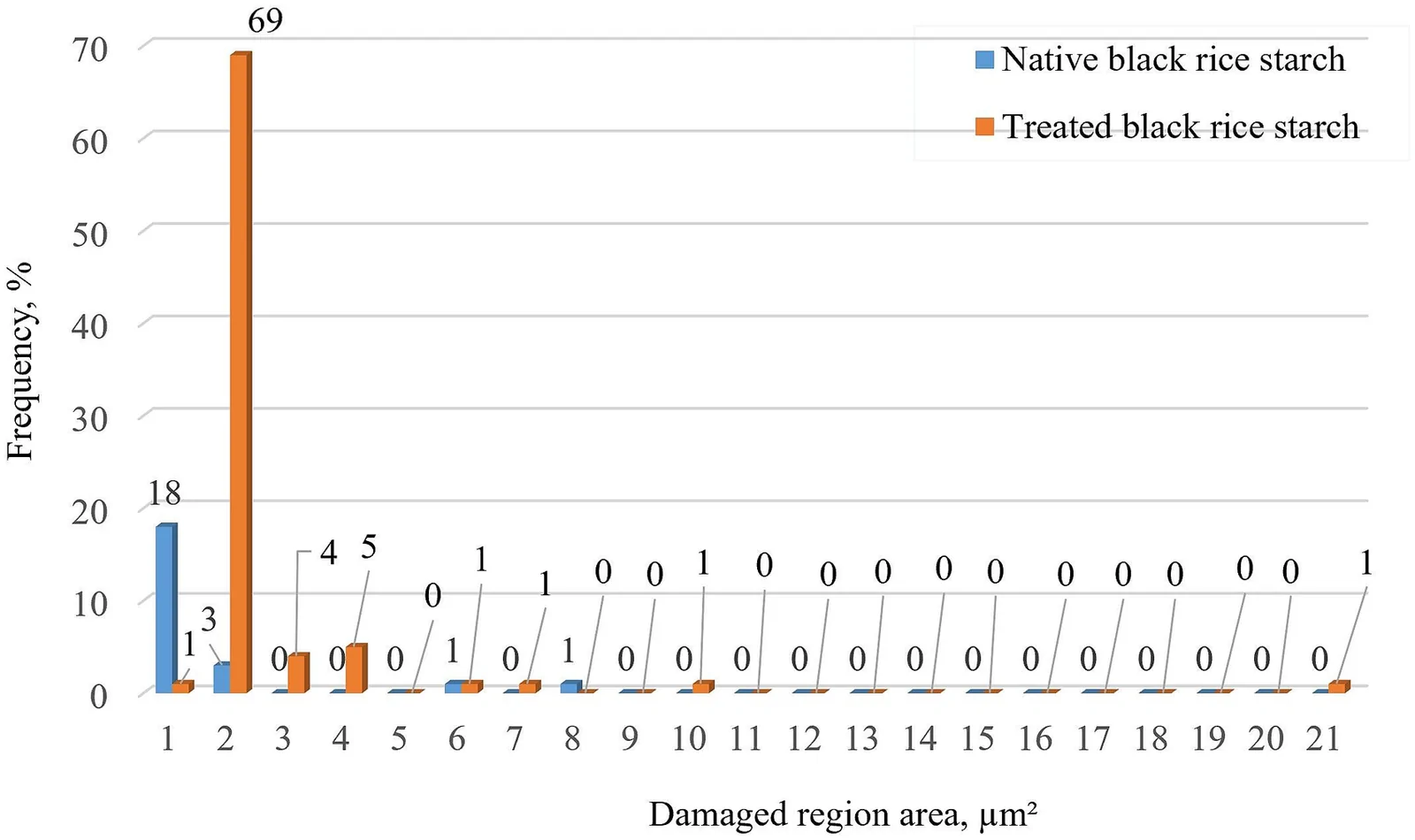
Representative histograms of damaged-region area distribution in starch granules isolated from brown and black rice flours before and after steam-assisted heat treatment (SAHT, 100 °C, 90 min). The histograms illustrate the contrasting effects of SAHT on the size distribution and frequency of damaged regions detected on starch granule surfaces. The complete set of histograms for all rice varieties is provided in Fig. 3.SM. (For interpretation of the references to colour in this figure legend, the reader is referred to the web version of this article.)

Native starches exhibited substantial variability in surface roughness. Red rice starch showed the highest Ra and Rq values (1254 and 1311 nm, respectively), indicating a heterogeneous and irregular surface, while brown rice starch had intermediate roughness, and black rice starch the lowest, corresponding to a smoother, more uniform surface. These differences reflect intrinsic cultivar-specific surface characteristics. Elevated roughness in red and brown rice starches likely arises from both compositional factors and mechanical effects during milling and starch isolation, which accentuate microscale surface heterogeneity. SAHT caused marked changes in starch surface topography, with effects strongly dependent on rice type. In brown rice starch, Ra and Rq decreased approximately four- to five-fold (414.1 → 91 nm; 512.2 → 120 nm), reflecting pronounced smoothing likely due to surface gelatinization and molecular reorganization, including swelling and partial collapse or fusion of surface features. Red rice starch showed a similar but less pronounced reduction in roughness. Although Ra and Rq decreased substantially, from 1254 to 552 nm (56.0%) and from 1311 to 646 nm (50.7%), respectively, they remained higher than in other treated samples, indicating incomplete surface homogenization. This may reflect greater internal heterogeneity or a reduced capacity of red rice starch granules to undergo uniform surface reorganization under SAHT conditions. In contrast, black rice starch showed an increase in Ra and Rq after SAHT (307 → 482 nm; 388 → 672 nm), indicating net surface roughening. This suggests that steam-assisted heating induced localized surface disruption, likely due to uneven water penetration, heterogeneous swelling, or formation of nanoscale irregularities. The distinct response of black rice starch may be associated with differences in its native molecular organization and overall flour composition. Overall, AFM results show that SAHT induces rice-type-dependent starch surface restructuring. Brown and red rice starches primarily undergo surface smoothing, associated with molecular rearrangement and partial gelatinization, whereas black rice starch exhibits increased nanoscale roughness. These observations complement FTIR, XRD, SEM, and image analysis, providing multi-scale evidence of starch reorganization. In brown and red rice, reductions in Ra and Rq align with increases in relative and total crystallinity, enhanced A-type crystallinity, and formation of *V*-type amylose–lipid complexes, collectively promoting surface homogenization. In black rice, increased roughness, despite moderate A-type crystallinity gains, reflects reduced *V*-type crystallinity and disruption of amylose–lipid complexes, leading to localized surface heterogeneity rather than uniform smoothing. Thus, AFM complements spectroscopic, diffraction, and microscopic analyses.

#### 3.1.4 Scanning electron microscopy

The SEM analysis revealed that SAHT induced substantial microstructural modifications in the starch fraction of brown, red, and black rice flours, although the extent and nature of these changes differed among rice types (Table 2). These alterations resulted from the combined action of heat and moisture and were reflected both in the morphology and in the size of the starch granules. Brown rice starch exhibited the most pronounced structural disruption, characterized by extensive granule collapse, surface fissures, and loss of the original granular architecture. This severe alteration was accompanied by the complete disappearance of clearly distinguishable starch granules, which were replaced by a collapsed, continuous matrix containing irregular cavities. Red rice starch also underwent significant modification, with a few residual granules remained identifiable after treatment. The maximum Feret diameter decreased markedly from 7.2 ± 1.4 μm in the native red rice flour to 2.7 ± 0.6 μm after SAHT, indicating substantial granule shrinkage and fragmentation. In contrast, black rice starch retained a comparatively smoother and more compact morphology, with fewer and less widely distributed surface defects than those observed in brown rice starch. Although the native black rice starch granules exhibited a maximum Feret diameter of 7.5 ± 1.6 μm, SAHT transformed the starch into a continuous gelatinized matrix, suggesting extensive molecular reorganization despite the lower degree of visible surface disruption. Overall, the SEM observations indicate distinct morphological responses of the three rice starches to SAHT, with brown rice starch showing the greatest surface modification and structural collapse, red rice starch exhibiting intermediate changes associated with granule size reduction, and black rice starch displaying the least apparent surface damage despite the formation of a gelatinized matrix.

**Table 2:** SEM-based assessment of starch characteristics.

Sample	SEM	Granule shape and size	Surface features	Granule arrangement	Structural integrity	Digestibility and potential use
Starch from untreated brown rice flour (Pintilii et al., 2025)		Starch granules are polygonal or spherical, with relatively uniform dimensions ranging from 5 to 10 μm. Maximum Feret diameter: 3.92 ± 1.45 μm	Surface textures display minor irregularities and indentations, indicative of milling process.	Granules exhibit moderate clustering but remain clearly distinguishable from one another.	The structure appears fragile, with a tendency to swell and break under stress.	–
Starch from brown rice flour subjected to steam-assisted heat exposure for 90 min		Native starch granules are no longer clearly distinguishable. A collapsed, continuous matrix with irregular cavities is observed. Apparent structural domains are several micrometers across. Wet heat treatment caused granule swelling, rupture, and fusion, masking original granule morphology.	Surface appears rough, uneven, and porous, with multiple round to elongated voids, ranging roughly from submicron to a few μm. Edges are softened and melted rather than sharp. Indicates leaching of amylose, and internal granule collapse during heating. Increased porosity and surface area are typical of gelatinized starch.	Granules are aggregated and fused into a continuous mass rather than individually packed. No clear inter-granular boundaries are visible. Suggests inter-granular bonding and coalescence during wet heating, forming a starch gel–like network upon drying.	Severely compromised fractured walls, internal cavities, and loss of native crystalline organization. Structure resembles a sponge-like or honeycomb matrix. Confirms near-complete gelatinization, with destruction of semicrystalline lamellae.	Porous, amorphous structure with disrupted crystallinity. Likely higher enzymatic digestibility and glycemic response due to easier enzyme penetration. Suitable for instant foods, thickeners, gluten-free baked goods and porridges. Porous matrix may also be useful for encapsulation, biodegradable films, or modified starch-based delivery systems.
Starch from untreated red rice flour (Pintilii et al., 2025)		Angular and polygonal granules are observed, generally smooth-edged, with sizes between 5 and 8 μm. Maximum Feret diameter: 7.2. ± 1.4 μm	Surface morphology is smooth and well-preserved across most granules.	Granules remain discrete, with minimal clustering or interaction.	Exhibits high mechanical resistance, suggesting a robust crystalline structure.	–
Starch from red rice flour subjected to steam-assisted heat exposure for 90 min		Irregular fragmented domains with remnants of swollen granules fused together. Some rounded features (∼2–5 μm) appear collapsed rather than intact. Indicates partial to extensive gelatinization. Granules initially swelled and then ruptured, losing their original polygonal/ oval rice starch morphology. Maximum Feret diameter: 2.7. ± 0.6 μm	Surface is highly rough, wrinkled, and fractured, with visible cracks and shallow pits. Compared to native starch, surfaces appear melted and uneven rather than smooth. Reflects amylose leaching, surface erosion, and thermal stress during wet heating. Cracks suggest dehydration shrinkage after gelatinization.	Granules appear aggregated and compacted, forming clusters rather than a continuous matrix. Some inter-granular boundaries are faintly visible, unlike a fully fused gel network. Suggests incomplete fusion of swollen granules-wet heat disrupted granules but did not fully homogenize them into a single matrix.	Moderately compromised: granules are deformed and fractured but not completely destroyed. Residual granular outlines persist in some regions. Indicates partial gelatinization, with destruction of crystalline regions but retention of some granular framework.	Rough, cracked, partially amorphous structure with residual granule fragments. Enhanced digestibility compared to native starch, but slightly lower than brown starch. Suitable for thickening agents, controlled-viscosity foods, noodles and pasta. Partial integrity may also favor moderate digestibility and slower glucose release.
Starch from untreated black rice flour (Pintilii et al., 2025)		Granules range from 6 to 12 μm in size, with both regular and irregular morphologies represented. Maximum Feret diameter: 7.5. ± 1.6 μm	Surface characteristics are smooth and indicative of a highly crystalline state.	Aggregation is limited, with granules mostly remaining well-separated.	Displays significant resistance to mechanical and structural disruption.	–
Starch from black rice flour subjected to steam-assisted heat exposure for 90 min		Starch granules were transformed in continuous gelatinized matrix with lost boundary definition and smooth, amorphous contours.	The granules appear rough and irregular in texture, which suggests some damage to the surface. This is consistent with the wet heat treatment, which likely leads to partial gelatinization and disruption of the granule's smooth surface. There are visible cracks and wrinkles, indicating that the structure has undergone thermal modification.	The granules are scattered, with no uniform arrangement. This scattered pattern might be due to the processing methods and isolation from the black rice flour. Some of the granules are clustered, while others appear isolated.	Structural integrity is partially compromised. The visible cracks, wrinkles, and uneven surface suggest that the starch granules have been altered, possibly undergoing some degree of gelatinization. Some granules retain their original shape.	The damage to the granules suggests that the flour is suitable for food products requiring controlled hydration and structural firmness, including extruded snacks and breakfast cereals. However, some of the granules may still have intact regions that could resist enzymatic breakdown.

#### 3.1.5 Image analysis

Analysis of the damaged-region area distributions revealed distinct modifications in the surface morphology of starches isolated from brown, red, and black rice flours following SAHT. Representative histograms illustrating the contrasting responses of brown and black rice starches are shown in Fig. 4a and b, whereas the complete set of histograms is provided in Fig. 3.SM.

The surface voids area distribution of starch granules from native and treated brown rice flour differs not only in their size frequency but also in distribution shape, skewness, and tail behavior, indicating treatment-induced modifications in granule surface microstructure. Native brown rice starch shows a positively skewed distribution with a long right tail, where most surface voids are small (≈1–3 μm^2^) but a few extend to much larger areas (up to ∼21 μm^2^), reflecting a heterogeneous morphology and intrinsic structural variability arising from uneven organization of amorphous and crystalline regions. In contrast, treated starch also exhibits positive skewness but with a markedly narrower, light-tailed distribution, characterized by a strong clustering of surface voids at ∼1 μm^2^ and a sharp decline in larger surface voids occurrences. Comparatively, the treated starch exhibited a narrower distribution characterized by a lower frequency of large surface defects and a more homogeneous distribution of surface features, indicating substantial modification of granule surface morphology following SAHT. The surface voids area distribution of red rice starch granules reveals clear treatment-induced changes in both frequency and distribution shape. Native red rice starch exhibits a positively skewed distribution with a moderately long right tail, characterized by a high frequency of small surface voids (≈1–2 μm^2^) alongside sporadic medium-sized surface voids extending to ∼11 μm^2^. This tail behavior reflects heterogeneous surface morphology and suggests the presence of intrinsic surface irregularities within native granules. In contrast, SAHT in red rice starch shows a strongly right-skewed but markedly narrower distribution, with surface voids overwhelmingly concentrated at ∼2 μm^2^ and a sharply truncated right tail. The near absence of larger surface voids indicates that larger surface defects were less frequently observed after treatment. Overall, treatment was associated with a shift from a moderately heavy-tailed distribution to a narrower distribution dominated by small surface defects, indicating a more homogeneous surface morphology after SAHT.

Native black rice starch displays a positively skewed surface voids area distribution with a pronounced right tail, where most voids are concentrated at small areas (≈1 μm^2^) but isolated surface voids extend to much larger sizes (up to ∼21 μm^2^). This long, heavy right tail indicates substantial heterogeneity in granule surface architecture, likely arising from localized structural discontinuities or fissures. Following treatment, the surface voids area distribution becomes sharply right-skewed yet highly compressed, with the vast majority of voids clustered at ∼2 μm^2^ and a strongly truncated right tail. The dramatic reduction in large surface voids suggests that SAHT was associated with a marked reduction in the occurrence of large surface defects, resulting in a more uniform distribution of surface defects. Thus, similar to red and brown rice starches, treatment converts black rice starch from a broad, heavy-tailed distribution to a narrow, light-tailed one, substantial modification of granule surface morphology and reduced structural variability following treatment.

A comparative analysis of the damaged-region area histograms of starch isolated from treated brown, red, and black rice flours (Fig. 3.SM) revealed systematic differences in their size distribution and microstructural reorganization induced by SAHT. Treated brown rice starch exhibited the highest number of small surface voids (0.054–0.811 μm^2^), with a count of 32, indicating pronounced susceptibility to the treatment. This increase in small surface defects indicates a greater degree of surface disruption following treatment. In contrast, red and black rice starches showed much lower counts in this range (8 and 1, respectively), suggesting a more compact granular structure or lower susceptibility to modification. For medium-sized surface voids (0.812–1.567 μm^2^), brown rice starch again had the highest counts, though differences were smaller. Red rice starch showed moderate voids numbers (7), while black rice starch remained minimal (1), suggesting a comparatively less disrupted surface morphology after treatment. In the larger surface voids ranges (1.568–6.865 μm^2^), brown rice starch maintained higher counts (1–9), indicating a greater extent of surface disruption relative to red and black rice starches. Red rice starch showed fewer large surface voids (1–5), and black rice starch exhibited very few. Black rice starch showed an even lower presence of large surface defects, indicating a comparatively less disrupted surface morphology after treatment. In the highest surface voids area ranges (7.623–15.951 μm^2^), brown rice starch continued to show the most voids, though counts dropped to a maximum of 3. Red rice starch had 1–2 large voids, and black rice starch maintained 0–1, indicating minimal structural modification. Overall, brown rice starch exhibited the highest frequency and broadest distribution of surface defects across all area classes, indicating a greater degree of surface modification following SAHT. Red rice starch showed intermediate characteristics, whereas black rice starch retained the lowest frequency of surface defects and the narrowest distribution of defect areas. These observations suggest that the three rice starches differed in their morphological response to SAHT, with brown rice starch showing the most pronounced surface alteration and black rice starch the least.

#### 3.1.6 Starch digestion

The data presented in Fig. 5 show clear changes in starch digestibility fractions following steam-assisted heat treatment (SAHT). In brown rice flour (BRF), easily digestible starch (EDS) decreased from 48.74% in the untreated sample to 29.08% after 90 min, whereas slowly digestible starch (SDS) increased from 27.44% to 36.87% and resistant starch (RS) from 23.82% to 34.05%. Red rice flour (RRF) exhibited a similar overall response, with EDS decreasing from 45.23% to 30.67%, SDS increasing from 32.11% to 39.09%, and RS increasing from 22.66% to 30.24%, despite some variation at intermediate treatment times. In black rice flour (BlRF), EDS decreased from 43.55% to 31.02%, while SDS and RS increased from 36.42% to 42.06% and from 20.03% to 26.92%, respectively. Thus, SAHT was associated with a redistribution of starch from the easily digestible fraction toward the slowly digestible and resistant fractions. The progressive increase in SDS may indicate the development of starch populations with lower enzymatic accessibility, potentially resulting from the structural reorganization observed during treatment. Statistical details are provided in Section 1.7.SM. The magnitude of these changes differed among the rice types. BRF exhibited the highest RS values throughout the treatment, followed by RRF and BlRF, whereas EDS remained highest in BlRF. This ranking is consistent with the greater increase in crystalline order observed for BRF, followed by RRF and BlRF (Table 1), suggesting an association between structural reorganization and reduced starch digestibility. However, **c**rystallinity alone cannot be used to establish the mechanism responsible for the increase in RS. The present XRD results demonstrate changes in long-range crystalline organization, particularly in A-type crystallinity, but do not independently quantify the contributions of amylose–lipid complex formation, starch retrogradation, or physical entrapment.

**Fig. 5:**
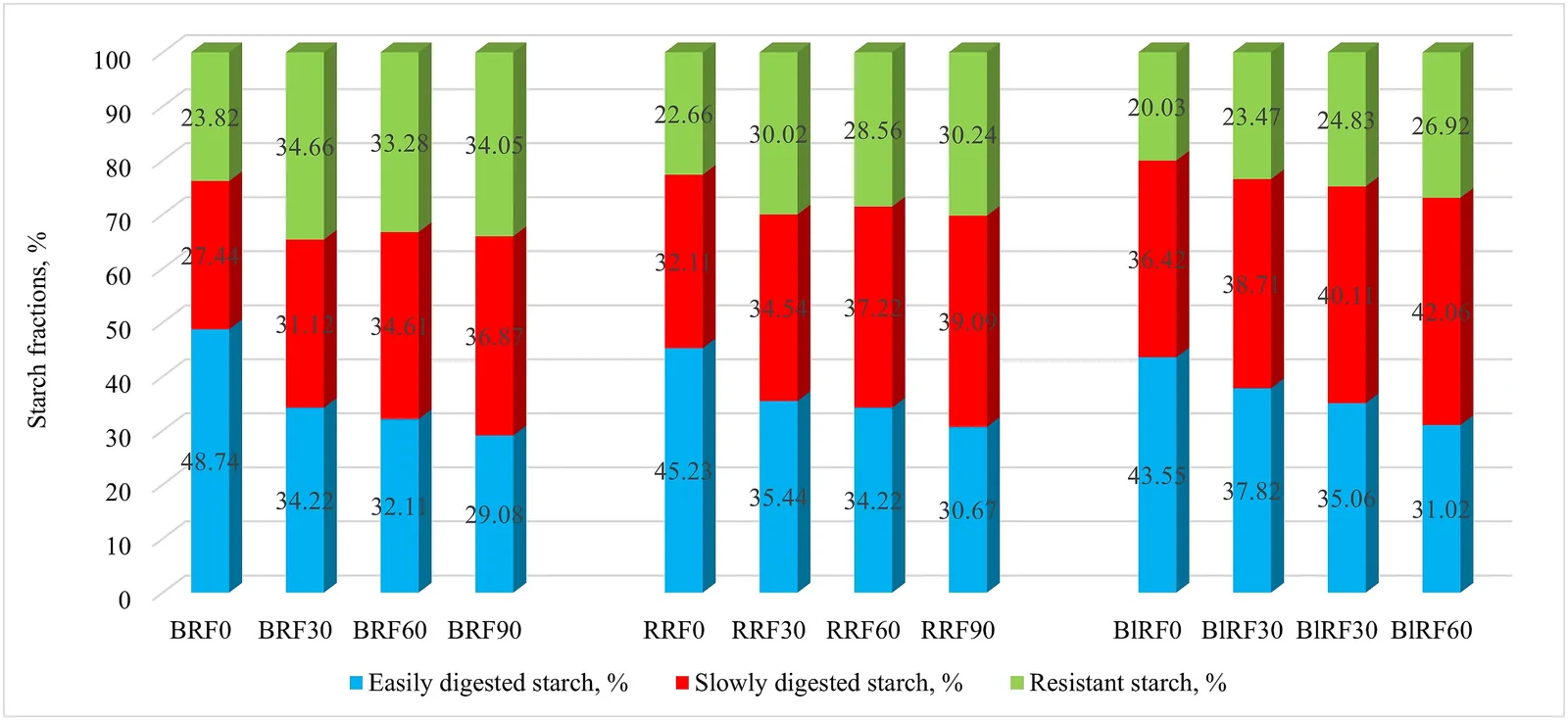
Changes in starch fraction composition of brown, red, and black rice flour during steam-assisted heat treatment: BRF0, BRF30, BRF60, BRF90 – starch isolated from brown rice flour subjected to steam-assisted treatment for 0, 30, 60 and 90 min; RRF0, RRF30, RRF60, RRF90 – starch isolated from red rice flour subjected to steam-assisted treatment for 0, 30, 60 and 90 min; BlRF0, BlRF30, BlRF60, BlRF90 – starch isolated from black rice flour subjected to steam-assisted treatment for 0, 30, 60 and 90 min. (For interpretation of the references to colour in this figure legend, the reader is referred to the web version of this article.)

Several mechanisms may therefore contribute concurrently to the observed digestibility response. The increase in A-type crystallinity indicates greater ordering of starch domains and may contribute to reduced enzyme accessibility. Changes in *V*-type crystallinity may also reflect modifications involving amylose–lipid associations; however, because amylose–lipid complex content was not independently quantified, the present data cannot establish the extent to which such complexes contributed to RS formation. Previous studies have shown that amylose–lipid inclusion complexes can reduce starch digestibility by limiting molecular mobility and accessibility to hydrolytic enzymes (Bienkiewicz & Kołakowska, 2004; Putseys et al., 2010). Similarly, cooling after treatment may have promoted some molecular reassociation or retrogradation, but retrograded starch was not directly quantified in the present study. Interactions involving proteins and other non-starch constituents could additionally influence enzyme accessibility, although these effects were not independently evaluated here. Accordingly, the increase in RS observed after SAHT should be interpreted as an experimental outcome associated with treatment-induced structural reorganization, rather than as evidence for a specific RS-forming mechanism. The present results establish a relationship between changes in starch crystalline structure and digestibility, while the relative contributions of amylose–lipid complex formation, recrystallization/ retrogradation, and physical restriction of enzyme access remain unresolved. Direct measurements of amylose–lipid complexes, retrograded starch, molecular mobility, and enzyme accessibility would be required to distinguish these mechanisms and quantify their respective contributions. In BRF, the decrease in *V*-type crystallinity occurred despite a substantial increase in total and A-type crystallinity. This observation further illustrates that changes in a single crystalline population cannot adequately explain the digestibility response. The concurrent increase in overall crystalline order and RS suggests that the net structural reorganization was associated with reduced starch susceptibility to enzymatic hydrolysis. Nevertheless, the present data do not allow attribution of this response to A-type crystallinity alone or to any individual molecular mechanism.

#### 3.1.7 Relationship between starch structure and digestibility

To integrate the observations obtained from spectroscopy, X-ray diffraction, microscopy, image analysis, and *in vitro* digestion, Table 3 summarizes the structural changes induced by SAHT and their association with the measured digestibility response. The combined results indicate that changes in starch surface morphology alone do not adequately explain the observed differences in enzymatic hydrolysis. Brown rice starch exhibited pronounced surface modification, including changes in nanoscale roughness and surface-defect distribution, while also showing the largest increase in long-range crystallinity and the highest resistant starch (RS) fraction. In contrast, black rice starch retained a higher proportion of surface defects but showed a smaller increase in long-range crystallinity and a lower RS fraction. Thus, the presence or development of surface defects was not consistently associated with greater enzymatic susceptibility across the rice varieties. Rather, the comparative results suggest that the digestibility response was more closely associated with the overall structural organization of the starch, particularly the extent of crystalline reorganization, than with surface morphology alone. Nevertheless, surface characteristics may still influence enzyme–starch contact and accessibility, and their contribution cannot be excluded. Because enzyme accessibility and diffusion, retrograded starch, amylose–lipid complex content, and physical entrapment within protein or dietary-fiber matrices were not independently measured, the present findings demonstrate an association between surface morphology, structural reorganization, and digestibility rather than a causal relationship.

**Table 3:** Integrative relationship between starch structural changes and digestibility after SAHT.

**Structural level or method**	**Main observation after SAHT**	**Interpretation supported by the present data**	**Association with digestibility**	**Important limitation**
FTIR short-range order	Relative short-range molecular order increased rapidly during the early treatment period, followed by only minor variation at longer treatment times	AHT was associated with increased local double-helical organization.	The development of local ordered structures was associated with the shift from rapidly digestible to slowly digestible and resistant starch fractions.	FTIR-derived RCD does not measure long-range crystallinity or enzyme accessibility.
XRD long-range order	Total and A-type crystallinity increased in all rice types, with the greatest increase in brown rice and a more moderate increase in black rice.	SAHT was associated with increased long-range crystalline organization.	The higher resistant starch fraction in brown rice occurred together with the largest increase in long-range crystallinity.	This association does not demonstrate that crystallinity alone caused the increase in resistant starch.
V-type diffraction features	V-type contributions changed differently among the rice types after treatment.	The changes are consistent with possible formation, alteration, or dissociation of amylose–lipid inclusion complexes, but these processes were not directly measured.	Changes in V-type structures may be associated with differences in starch hydrolysis.	Amylose–lipid complex content was not independently quantified.
AFM surface morphology	Brown and red rice starches showed reduced surface roughness, whereas black rice starch showed increased roughness after SAHT.	SAHT induced variety-dependent nanoscale surface restructuring.	Surface morphology may influence enzyme–starch contact and initial enzyme accessibility.	AFM measures the dried, isolated surface and does not directly measure enzyme diffusion or hydrolysis.
SEM morphology and surface-defect analysis	Brown rice showed the greatest visible collapse and surface-defect modification; red rice showed intermediate changes; black rice showed fewer visible defects.	SAHT produced variety-dependent microscale morphological modification.	Surface defects alone did not explain the digestibility pattern, since brown rice showed substantial surface modification but also the highest resistant starch fraction.	The observed features may be influenced by starch isolation, drying, fracture, and microscopy preparation; they should not be interpreted as intrinsic pores.
*In vitro* digestibility	Rapidly digestible starch decreased, whereas slowly digestible and resistant starch fractions increased. The resistant starch ranking was brown rice > red rice > black rice.	SAHT shifted starch toward less rapidly hydrolyzed fractions under the applied assay conditions.	The digestibility response was associated with structural reorganization at molecular, crystalline, and morphological levels.	The assay does not determine the individual contributions of crystallinity, retrogradation, amylose–lipid complexes, protein, fiber, or enzyme diffusion.
Overall interpretation	Structural and digestibility changes occurred simultaneously but differed among rice varieties.	Digestibility appears to be associated with the combined effects of molecular order, crystalline organization, matrix composition, and surface morphology.	Brown rice provides the clearest association between increased long-range order and increased resistant starch.	The proposed mechanism is correlational because the individual pathways were not independently tested.

### 3.2 Protein characterization

#### 3.2.1 FTIR spectra

The treatment-induced changes in individual secondary-structure components are presented in Fig. 6a–d. Brown rice showed a pronounced increase in β-sheet content (Fig. 6b), accompanied by marked decreases in β-turn (Fig. 6c) and random coil (Fig. 6d), whereas the increase in α-helix was comparatively smaller (Fig. 6a). ANOVA results are presented in Table 1. In native flours, red rice had the highest α-helix content (40.56%), whereas brown rice had the lowest (22.45%). Significant differences (*p* < 0.05) were observed for α-helix, β-sheet, and β-turn contents, whereas random coil content did not differ significantly between red and black rice. Red and black rice differed from brown rice in the distribution of individual secondary-structure components, particularly in α-helix and β-sheet contents. The combined α-helix + β-sheet values were 74.89%, 64.27%, and 59.99% for red, black, and brown rice, respectively, and are reported here only as an auxiliary descriptor. These differences may reflect the distinct overall composition of the rice flours, including protein, lipid, fiber, and phenolic constituents (Table 1.SM). Although protein–polyphenol interactions have been reported to influence protein structure (Ozdal et al., 2013), their contribution to the differences observed among the present rice flours was not directly evaluated. Therefore, the differences in protein secondary structure are interpreted primarily as variety-dependent responses associated with the overall flour matrix rather than being attributed to a specific polyphenol-mediated mechanism.

**Fig. 6:**
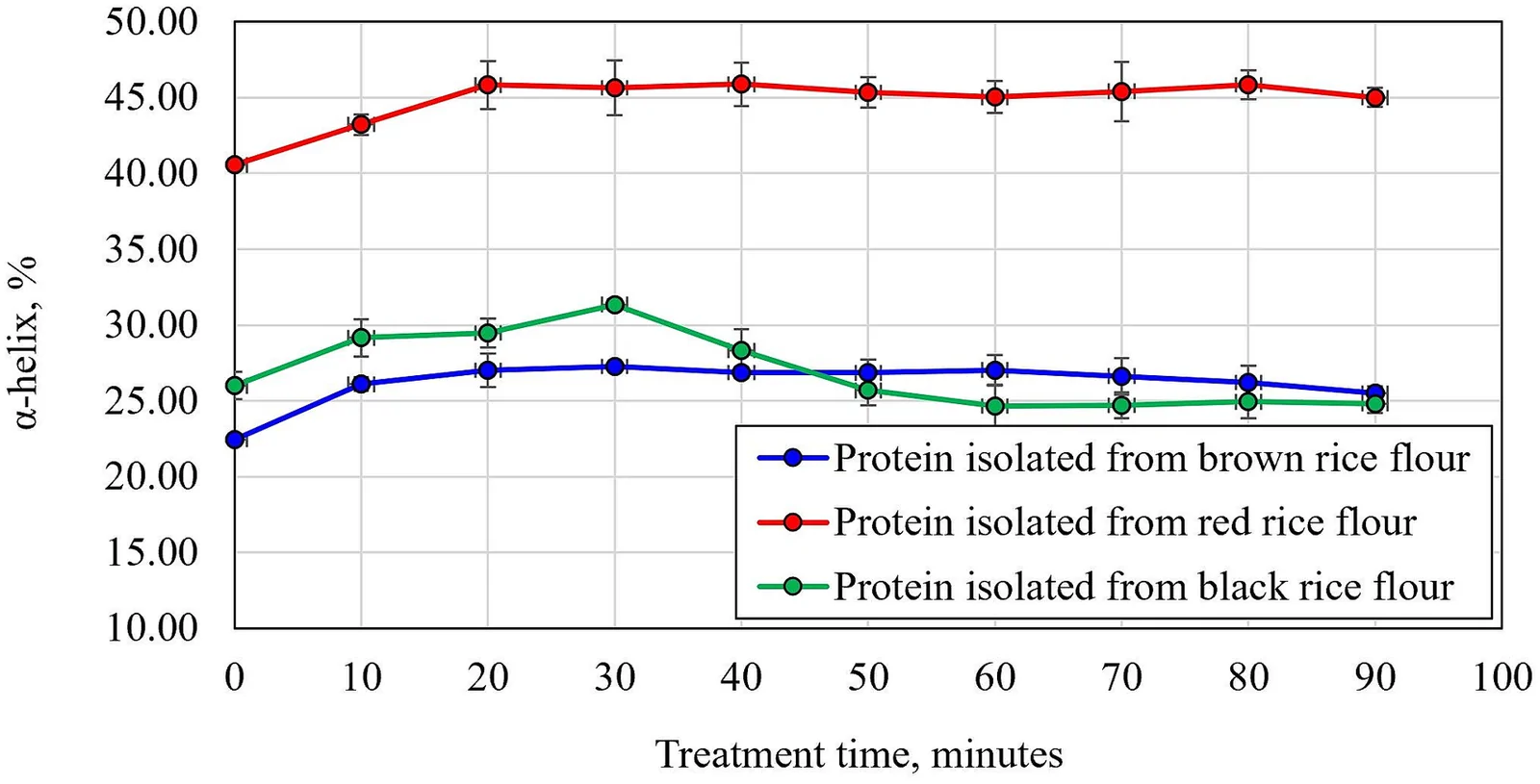

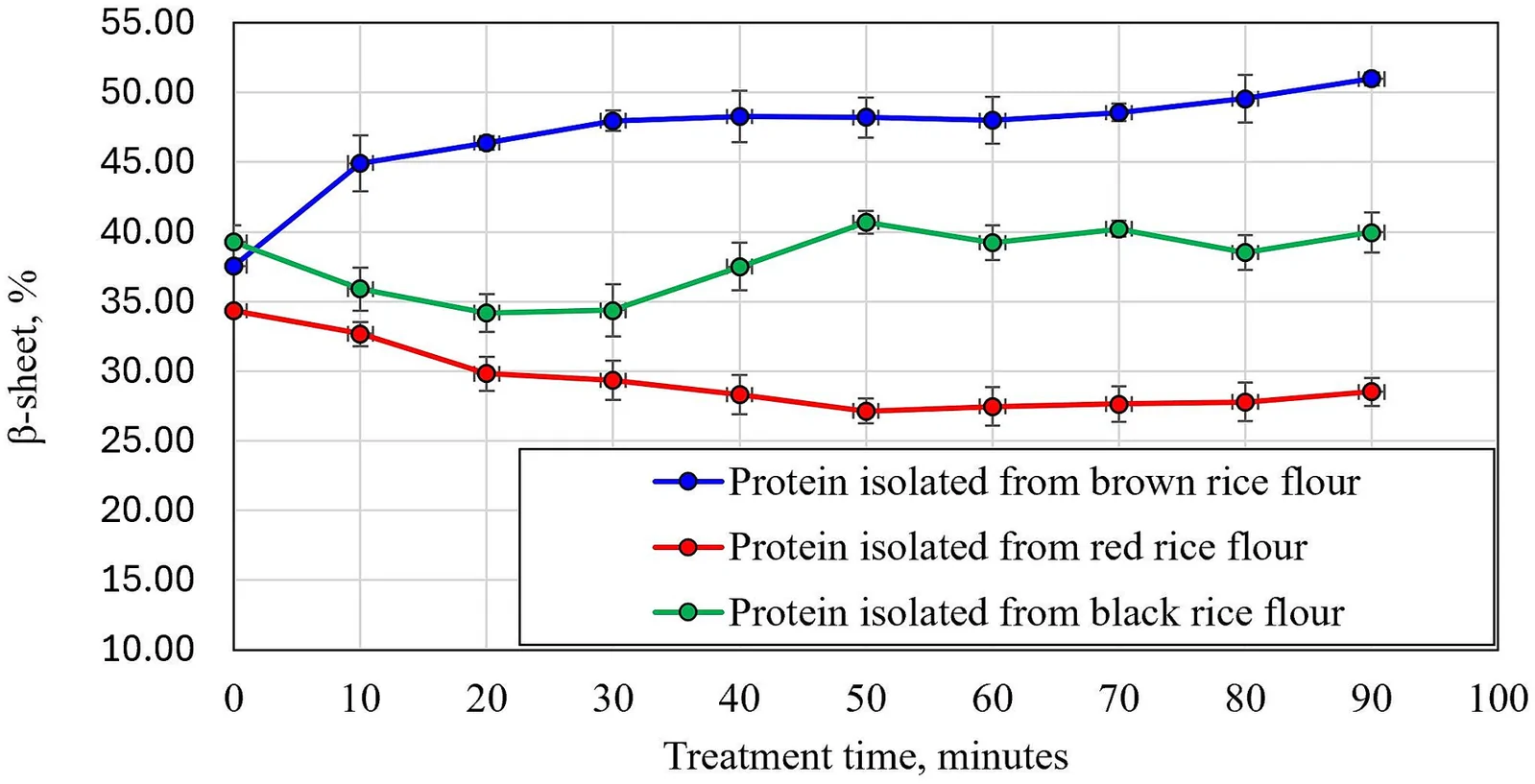

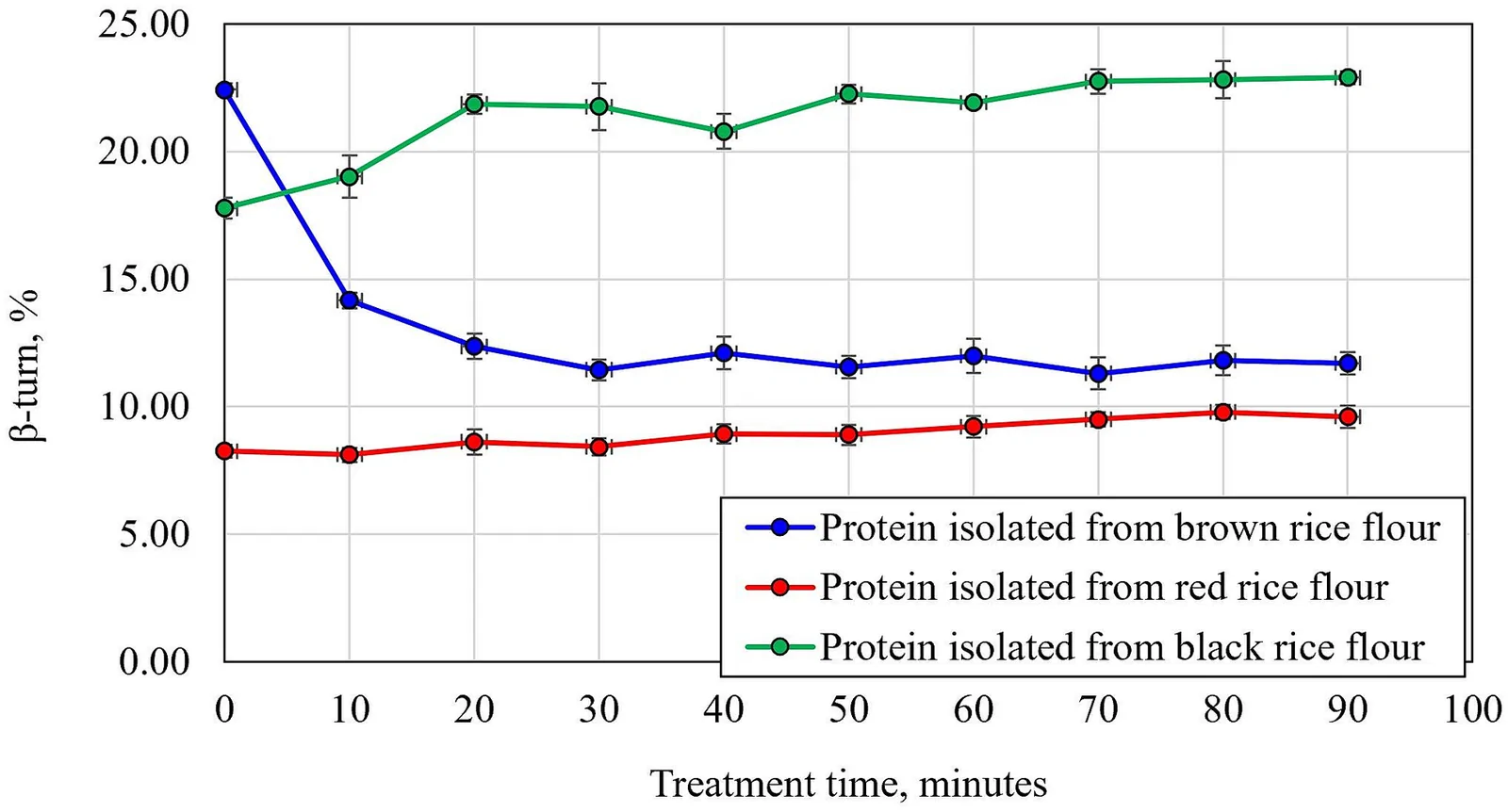

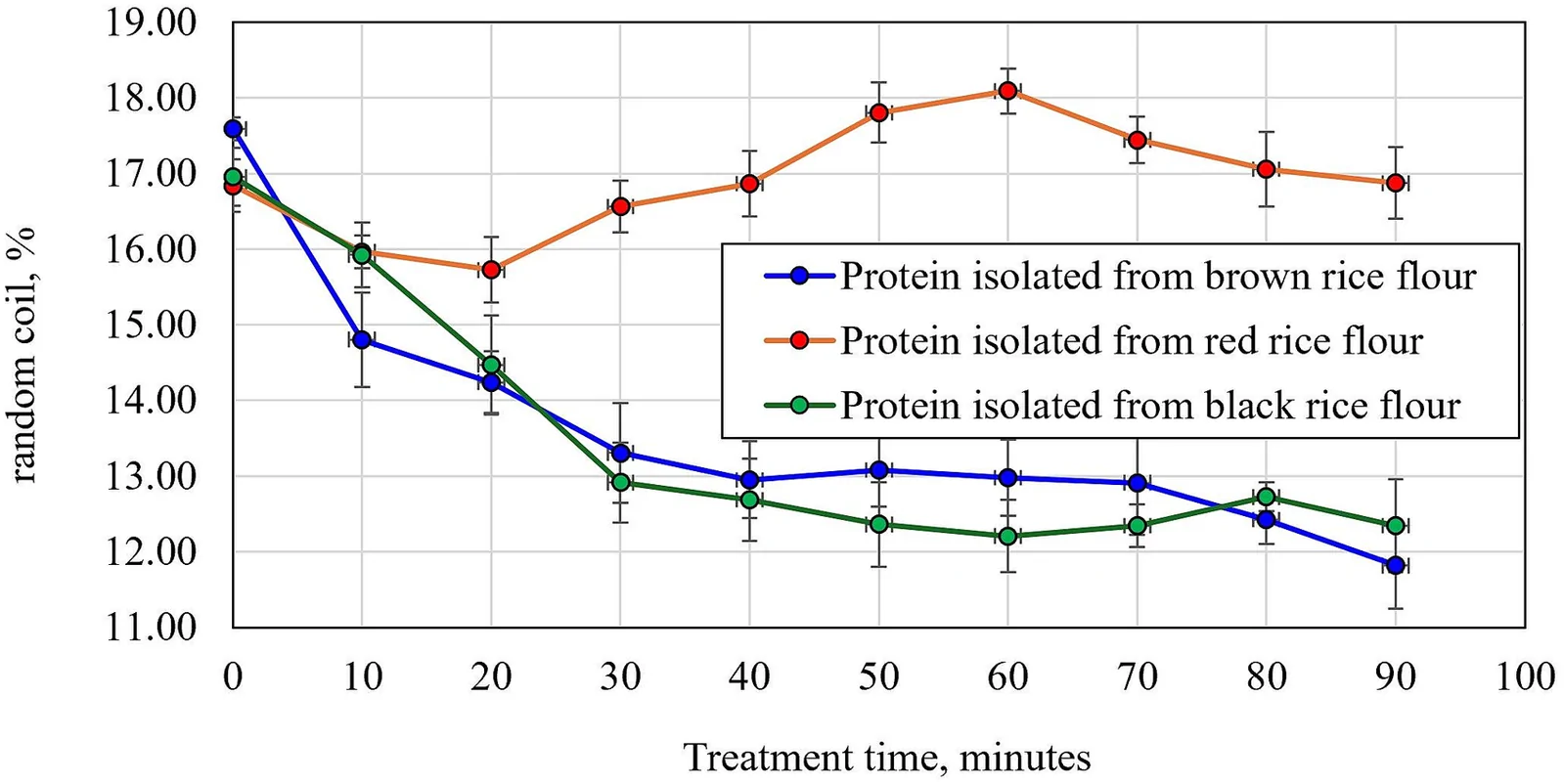
Changes in the secondary structure of proteins isolated from brown, red, and black rice flours during SAHT: (a) α-helix, (b) β-sheet, (c) β-turn, and (d) random coil. Values represent the mean ± SD. (For interpretation of the references to colour in this figure legend, the reader is referred to the web version of this article.)

At the end of SAHT, significant differences in protein secondary structure were observed among brown, red, and black rice flours (Table 1), indicating distinct treatment responses. Brown rice exhibited the most pronounced conformational reorganization. Although α-helix content increased by 13.63%, the increase in β-sheet was substantially more (+35.78%), rising from 37.54% in the native flour to 50.97% after 90 min of treatment. This β-sheet enrichment was accompanied by marked decreases in β-turn (−47.77%) and random coil (−32.80%) contents. Thus, the principal structural change in brown rice was not simply an increase in the combined α-helix + β-sheet fraction, but a redistribution of secondary structure characterized by a disproportionate increase in β-sheet accompanied by substantial decreases in β-turn and random coil structures. The increase in β-sheet is consistent with heat-induced protein association, aggregation, or refolding into β-sheet-rich structures. However, because FTIR analysis cannot unambiguously distinguish aggregation from refolding, these results are interpreted as evidence of substantial conformational reorganization rather than definitive proof of aggregation. The simultaneous reduction in β-turn and random coil contents further suggests decreased conformational flexibility and formation of a more compact protein structure. In contrast, red rice flour showed minor changes, indicating greater resistance to heat-induced conformational changes. The α-helix content increased slightly, β-sheet content decreased, and β-turn content increased, whereas random coil remained essentially unchanged. Although the combined α-helix + β-sheet value changed only slightly (−1.83%), the individual changes in α-helix and β-sheet indicate a redistribution between these secondary structures rather than preservation of an unchanged structural state. The high initial α-helix content in native red rice may be associated with its greater resistance to heat-induced conformational changes. For black rice flour, SAHT induced selective but moderate changes. The α-helix content decreased slightly, while β-sheet remained largely unchanged. The most notable effects were an increase in β-turn and a decrease in random coil structures. Although the combined α-helix + β-sheet content changed minimally (+0.76%), the marked decrease in random coil and increase in β-turn indicate substantial reorganization of the flexible regions, while the relatively small changes in α-helix and β-sheet suggest preservation of the major secondary-structure framework.

The evolution of rice protein secondary structure during SAHT is likely influenced by interactions with non-protein components. Red and black rice flours, richer in phenolics, fats, and ash, exhibited distinct initial distributions of α-helix, β-sheet, β-turn, and random coil structures, potentially reflecting protein–polyphenol (Ozdal et al., 2013) and protein–lipid interactions (Li et al., 2023). In contrast, brown rice flour showed the most pronounced conformational reorganization, characterized particularly by a marked increase in β-sheet accompanied by decreases in β-turn and random coil contents. These changes are consistent with heat-induced restructuring toward a more compact protein conformation, although FTIR alone cannot distinguish unequivocally between aggregation and refolding. Overall, the results demonstrate that SAHT affects protein secondary structure in a flour-dependent manner, likely reflecting differences in protein composition and interactions with non-protein components. To our knowledge, few studies have examined the impact of SAHT on protein structure in pigmented rice flours.

#### 3.2.2 Quantification of free -SH, total -SH and -SS bond content in rice protein isolates

Cysteine residues in proteins contain free thiol groups, which are reactive and capable of participating in chemical interactions, oxidation, or binding with other molecules. These groups can be oxidized to form disulfide bonds, playing a key role in maintaining the tertiary and quaternary structures of proteins. Table 4 summarizes the alterations in free –SH, total –SH, and –SS contents in rice protein isolates following SAHT.

**Table 4:** The changes of free -SH, total -SH and -SS content in rice protein isolates from rice flour subjected to steam-assisted heat exposure.

		Brown rice protein isolate			Red rice protein isolate			Black rice protein isolate		
		Native state	Exposure for 60 min	Exposure for 90 min	Native state	Exposure for 60 min	Exposure for 90 min	Native state	Exposure for 60 min	Exposure for 90 min
Free sulfhydryl (free -SH)	amount, mM/g	0.41 ± 0.01^bα^	0.71 ± 0.03^aα^	0.20 ± 0.01^cγ^	0.36 ± 0.02^cβ^	0.66 ± 0.01^aβ^	0.44 ± 0.01^bβ^	0.32 ± 0.01^cγ^	0.63 ± 0.01^bβ^	0.89 ± 0.02^aα^
	variation after treatment, %	–	+ 73.17	−51.22 −71.83^⁎^	–	+83.33	+22.22 −33.33^⁎^	–	+96.88	−178.13 +41.27^⁎^
Total sulfhydryl (total -SH)	amount, mM/g	0.54 ± 0.02^cβ^	0.92 ± 0.04^bγ^	1.03 ± 0.01^aγ^	0.60 ± 0.02^cβ^	1.18 ± 0.01^bβ^	1.28 ± 0.01^aβ^	1.00 ± 0.05^cα^	1.42 ± 0.05^bα^	1.93 ± 0.01^aα^
	variation after treatment, %	–	+70.37	+90.74 +11.96^⁎^	–	+96.67	+113.33 +8.48^⁎^	–	+42.00	+93.00 +35.92^⁎^
Disulfide (-SS)	amount, mM/g	0.13 ± 0.01^cγ^	0.21 ± 0.04^bγ^	0.83 ± 0.01^aβ^	0.24 ± 0.01^cβ^	0.52 ± 0.01^bβ^	0.84 ± 0.01^aβ^	0.68 ± 0.02^cα^	0.80 ± 0.05^bα^	1.04 ± 0.02^aα^
	variation after treatment, %	–	+61.54	+538.46 +295.24^⁎^	–	+116.67	+250.00 +61.54^⁎^	–	+17.65	+52.94 +30.00^⁎^

Data are presented as mean ± standard deviation (*n* = 3). Values with different letters a, b, and c in the same line for the same rice type are significantly different (*p* < 0.05). Values with different letters α, β, and γ in the same line, for different rice flour types and the same treatment are significantly different (*p* < 0.05). * the values are reported to 60 min.

SAHT induced pronounced, time-dependent changes in the thiol–disulfide balance of brown rice protein isolate, reflecting sequential unfolding, thiol exposure, and oxidative cross-linking. In the native protein, free -SH content was low (0.41 mM/g), indicating limited cysteine accessibility. After 60 min, free -SH increased by 73.17% relative to the native protein, and total sulfhydryl content rose by 70.37% relative to the native protein, consistent with partial unfolding and exposure of buried thiol groups. Extending the treatment to 90 min caused a sharp decrease in free -SH (−51.22% relative to the native) and a more than five-fold increase in disulfide bonds (+538.46% relative to the native protein), indicating extensive thiol oxidation. Total -SH content continued to increase (+90.74% relative to the native protein), suggesting enhanced extractability of sulfur-containing residues due to unfolding and aggregation. Overall, these results support a two-stage mechanism in which initial thiol exposure is followed by oxidative cross-linking, leading to the formation of a rigid, disulfide-stabilized protein network with implications for solubility, gelation, and thermal stability.

SAHT induced significant, time-dependent changes in the thiol–disulfide balance of red rice protein isolate, reflecting progressive unfolding followed by partial oxidative restructuring. Native red rice protein exhibited low free -SH content (0.36 mM/g), indicating limited cysteine accessibility within a compact structure. After 60 min, free -SH increased by 83.33% relative to the native protein, consistent with thiol exposure during unfolding. At 90 min, free -SH decreased by 33.33% relative to 60 min but remained 22.22% above the native protein level, indicating partial thiol oxidation. Total -SH content increased steadily, by 96.67% relative to the native protein after 60 min and by 113.33% relative to the native protein after 90 min, reflecting enhanced accessibility of sulfur-containing residues. Disulfide bonds increased substantially, by 116.67% relative to the native protein after 60 min and by 250.00% relative to the native protein after 90 min, confirming thiol oxidation and covalent cross-linking. These results suggest a two-stage process of initial thiol exposure followed by controlled disulfide formation, with retention of elevated free -SH indicating a moderated aggregation behavior and balanced redox state in red rice protein. Compared with brown and red rice proteins, black rice protein showed the strongest response to prolonged heating, indicating higher susceptibility to thermal and oxidative restructuring. Native black rice protein contained 0.32 mM/g free -SH, reflecting limited cysteine exposure. After 60 min, free -SH increased by 96.88% relative to the native protein, indicating exposure of buried sulfhydryl groups. After 90 min, free -SH remained elevated, showing an increase of 41.27% relative to the 60 min sample and 178.13% relative to the native protein. This continuous rise indicates that thiol exposure exceeded oxidation, suggesting sustained unfolding and generation of accessible cysteine residues during aggregation. Total -SH content increased by 42.00% relative to the native protein after 60 min and by 93.00% relative to the native protein after 90 min, confirming enhanced accessibility of sulfur-containing residues. Disulfide bonds increased moderately, by 17.65% relative to the native protein after 60 min and by 52.94% relative to the native protein after 90 min, less than in brown and red rice proteins, indicating many thiols remained reduced. Overall, black rice protein unfolding dominates sulfur bond evolution, yielding high thiol availability and suggesting enhanced flexibility, reactivity, and functional versatility in thermally processed foods. The evolution of free -SH, total -SH, and disulfide (-SS) bonds during SAHT closely mirrors changes in rice protein secondary structure (Fig. 6, Table 1), indicating a coupled covalent–conformational response to heating. In brown rice, extensive thiol exposure and pronounced disulfide formation coincided with increased α-helix and, particularly, β-sheet contents and reduced β-turn and random coil contents, supporting substantial conformational reorganization toward a more compact, disulfide-stabilized structure. Red rice shows a more balanced thiol–disulfide response, consistent with the relatively moderate redistribution among individual secondary-structure components, including an increase in α-helix and a decrease in β-sheet. In black rice, the sustained rise in free and total -SH, along with moderate disulfide formation, aligns with preservation of α-helix and β-sheet structures and reorganization of disordered regions into β-turns, indicating a structurally resilient protein framework.

Collectively, these findings indicate that sulfur-bond chemistry and secondary-structure evolution were associated with the variety-dependent protein response during SAHT. The differences may reflect the distinct composition and molecular organization of the rice flour matrices. However, the present experiments do not allow the specific contribution of phenolic or lipid interactions to protein flexibility, redox behavior, or thermal stability to be established. To our knowledge, no studies have reported changes in free –SH, total –SH, or disulfide (-SS) contents of rice protein isolates under similar treatment. Results on protein hydrophobicity (Section 1.6.SM) further support the trends observed in FTIR analysis and the quantification of free –SH, total –SH, and -SS contents.

### 3.3 PCA analysis

Principal component analysis (PCA) clearly distinguished pigmented rice flours based on starch relative crystallinity and protein secondary structure, revealing marked structural changes induced by SAHT (Fig. 7a). In the native state, PC1 and PC2 together explained 99.68% of the total variance, with PC1 alone accounting for 94.67% (Table 3.SM), reflecting strong correlations among structural parameters and the small sample size. PC1 primarily captured variations in starch crystallinity closely associated with protein conformational features, whereas PC2 contributed minimally (5.01%), indicating minor protein secondary structure differences among native flours. After SAHT, cumulative variance explained by PC1 and PC2 decreased to 90.12%, with variance more evenly distributed between PC1 (48.66%) and PC2 (41.45%), suggesting additional independent sources of variability arising from treatment-induced changes in starch order and protein structure. Directional shifts of individual flours in PCA space further demonstrate that the magnitude and nature of these structural transformations are variety dependent. Overall, PCA highlights how SAHT modifies both the extent and relationships of structural attributes, while recognizing that observed patterns reflect correlations rather than causal mechanisms.

**Fig. 7:**
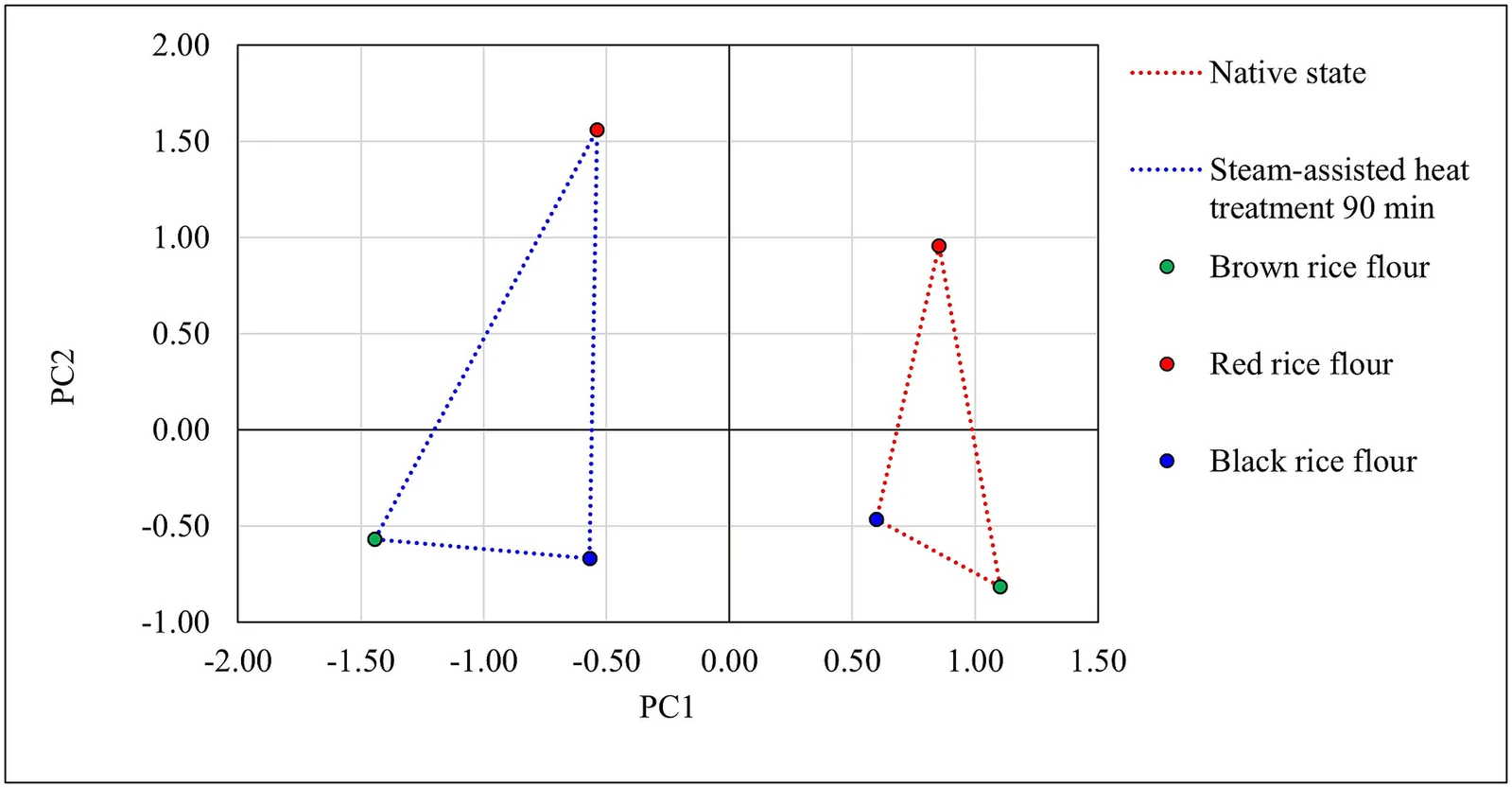

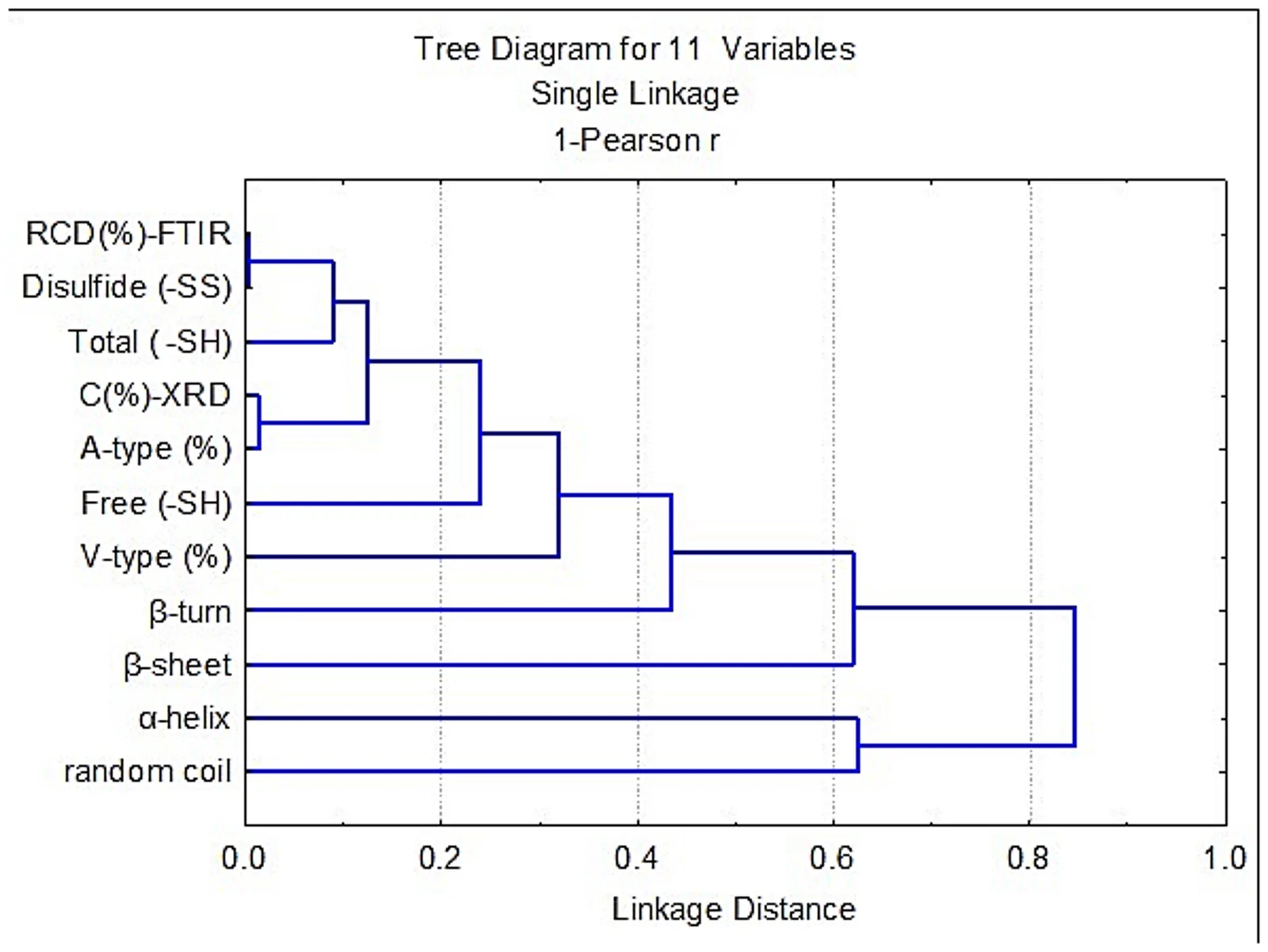
PCA analysis and hierarchical clustering of starch-protein interactions and structural parameters in native and steam-assisted heat treatment of brown, red, and black rice flours: a.Correlations between principal components PC2 and PC1 associated with the starch relative crystallinity degree and protein secondary structure in native and steam-assisted heat treatment of brown, red and black rice flours; b. Hierarchical clustering of starch-protein interactions based on structural parameters in native and steam-assisted heat treatment of brown, red and black rice flours. (For interpretation of the references to colour in this figure legend, the reader is referred to the web version of this article.)

Hierarchical cluster analysis using single linkage and 1–Pearson correlation (Fig. 7b) revealed clear groupings among structural parameters. Starch-related variables, including relative crystallinity from FTIR and XRD, and A-type crystallinity, clustered closely with protein thiol and disulfide parameters (total –SH, free –SH, and disulfide –SS), indicate strong associations within the starch–protein matrix. In contrast, protein secondary structural elements (β-sheet, α-helix, β-turn, random coil) formed a separate cluster, suggesting that secondary structure composition varies more independently of starch crystallinity. The relative distance of random coil and α-helix reflects higher flexibility and sensitivity to processing-induced unfolding. Overall, clustering highlights correlations between starch crystalline order and protein chemical bonding, while recognizing that these associations reflect coordinated patterns rather than causal relationships.

### 3.4 Potential technological implications and limitations

The structural relationships identified by the multivariate analysis provide a basis for considering the potential functional and industrial implications of SAHT. The close clustering of starch crystallinity parameters with thiol–disulfide variables suggests coordinated changes in starch molecular organization and protein chemical bonding. Although these statistical associations do not establish causality, they indicate that SAHT modifies multiple components of the flour matrix simultaneously, which may influence its behavior during subsequent food processing.

From a technological perspective, the structural modifications induced by SAHT may influence the functional properties of rice flours, which is particularly relevant for gluten-free applications. Brown rice flour exhibited the most pronounced starch and protein modifications, together with higher levels of slowly digestible and resistant starch, suggesting potential for applications requiring enhanced nutritional functionality and tailored processing characteristics. In contrast, black rice flour showed less disrupted starch morphology and greater protein stability, indicating better preservation of structural integrity, while red rice flour displayed an intermediate response to treatment. Nevertheless, these potential functional implications require direct validation because pasting viscosity, water-holding capacity, gel strength, gelatinization behavior, and rheological properties were not measured in the present study. Although starch digestibility and the proportions of rapidly digestible, slowly digestible, and resistant starch were evaluated, these *in vitro* measurements do not directly establish postprandial glycemic response. Such a processing–structure–function approach would help optimize SAHT conditions for specific applications and strengthen the translational potential of SAHT-treated pigmented rice flours. These possibilities require validation in real food systems and should not be interpreted as demonstrated product benefits from the present data alone.

## 4 Conclusions

Steam-assisted heat treatment induces clear, variety-dependent structural modifications in both starch and protein. In starch, brown and red rice exhibit pronounced reorganization, with substantial increases in crystallinity driven by both amylopectin recrystallization and amylose–lipid complex formation. In contrast, black rice starch shows a more moderate increase in crystallinity, dominated by amylopectin recrystallization and accompanied by a reduction in *V*-type structures, resulting in a denser and more compact architecture. Overall, these changes reflect a shift toward more ordered crystalline domains, particularly A-type structures, which impose steric and diffusional constraints on enzymatic hydrolysis and may promote the formation of resistant starch fractions. Protein responses mirror this variability: brown rice proteins form rigid, disulfide-stabilized networks, red rice proteins maintain moderate structural stability, and black rice proteins retain greater flexibility through reorganization of disordered regions. Multivariate analyses (PCA and hierarchical clustering) confirm coordinated, variety-specific reorganization of starch and protein features, highlighting the importance of starch–protein interactions in processing behavior. Although nutritional outcomes were not directly measured, the structural evolution suggests a potential increase in resistant starch, particularly in brown and red rice flours.

A limitation of the present SAHT configuration is the absence of direct measurement of relative humidity inside the ventilated oven. The amount of water placed in the chamber and the final flour moisture content provide information about water availability and net moisture uptake, but they do not establish the instantaneous relative humidity, steam concentration, or humidity uniformity throughout the chamber. Consequently, the present treatment should be interpreted as a standardized moist-air exposure under the specific equipment and operating conditions used in this study, rather than as a fully controlled steam-treatment protocol. Although the final moisture contents were similar among the rice flours, equivalent final moisture does not necessarily indicate identical moisture histories during heating. Differences in moisture absorption rate, local temperature, evaporation, and sample position may have affected the extent and timing of structural reorganization. Future studies should employ a sealed or semi-sealed treatment chamber equipped with calibrated temperature and RH sensors positioned near the sample. Controlled steam injection, continuous recording of temperature and RH, and reporting of sample moisture at several treatment times would allow the process to be defined by measurable atmospheric parameters. Interlaboratory comparison should also include a standardized sample mass, layer thickness, pan geometry, air-flow condition, and cooling protocol.

The present study also has limitations regarding the direct assessment of nutritional and technological functionality. Nutritional outcomes and functional properties were not directly measured; therefore, the observed structural changes, including the potential increase in resistant starch, particularly in brown and red rice flours, should not be interpreted as direct evidence of nutritional improvement or enhanced technological performance. The potential contributions of phenolic compounds, lipids, and other non-starch constituents to the observed structural responses were not independently evaluated. Therefore, the present findings do not establish a specific polyphenol-mediated mechanism, and further targeted studies are required to clarify the roles of individual flour constituents in the structural response to SAHT. Future studies should integrate functional property measurements, such as pasting, rheological, water absorption, swelling, and textural analyses, with thermal and digestibility-related assessments to establish robust processing–structure–function relationships.

## CRediT authorship contribution statement

**C. Pintilii:** Methodology, Investigation. **L. Mihaly Cozmuta:** Validation, Software, Formal analysis. **F. Popa:** Methodology, Investigation. **C. Bîrleanu:** Methodology, Investigation. **M. Pustan:** Methodology, Investigation. **M. Baia:** Methodology, Investigation. **K. Magyari:** Methodology, Investigation. **Z. Szakacs:** Methodology, Investigation. **C. Nicula:** Methodology, Investigation. **A. Peter:** Methodology, Investigation. **A. Mihaly Cozmuta:** Writing – original draft, Methodology, Investigation.

## Ethical statements

The work did not involve studies in humans or animals.

## Funding sources

This research did not receive any specific grant from funding agencies in the public, commercial, or not-for-profit sectors.

## Declaration of competing interest

The authors declare that they have no known competing financial interests or personal relationships that could have appeared to influence the work reported in this paper.

## Data Availability

Data will be made available on request.
