# Supplementary material for: Structural changes in starch and protein of brown, red, and black rice flours induced by steam-assisted heat treatment
Source: Food Chem X. 2026 Oct 27;40:104688. doi: 10.1016/j.fochx.2026.104688 (PMC13742231; doi:10.1016/j.fochx.2026.104688)
Supplement: Supplementary file 1 [file mmc1.docx]

**Structural changes in starch and protein of brown, red, and black rice flour induced by**

**steam-assisted heat treatment**

C. Pintilii^1^, L. Mihaly Cozmuta^1^, F. Popa^2^, C. Bîrleanu^3^, M. Pustean^3^, M. Baia^4,5^,

K. Magyari^5^, Z. Szakacs^1^, C. Nicula^1^, A. Peter^1^, A. Mihaly Cozmuta^1*^

^1^ Technical University of Cluj-Napoca, North University Center of Baia Mare, Victoriei Str. 76, Baia Mare, Romania*;* [**ancamihalycozmuta@gmail.com*](mailto:*ancamihalycozmuta@gmail.com)

^2^ Technical University of Cluj-Napoca, Department of Materials Science and Engineering,

Blv. Muncii 103-105, 400641, Cluj-Napoca, Romania ^3^ Technical University of Cluj-Napoca, Department of Mechanical Systems Engineering,

Blv. Muncii 103-105, 400641, Cluj-Napoca, Romania

^4^ Faculty of Physics, Babes-Bolyai University, Mihail Kogalniceanu Str. 1, 400084, Cluj-Napoca, Romania

^5^ Interdisciplinary Research Institute on Bio-Nano-Sciences, Babes-Bolyai University,

Treboniu Laurian Str. 42, 400271, Cluj-Napoca, Romania

**1.SM. Materials and methods**

***1.1.SM. Proximate analysis of rice flour types*** (Pintilii et al., 2025)

Table 1.SM. Proximate analysis of the investigated rice flour types

| Parameter | Brown rice flour | Red rice flour | Black rice flour |
| --- | --- | --- | --- |
| Moisture, % | 10.78 ± 0.13^b^ | 12.00 ± 0.18^a^ | 12.36 ± 0.20^a^ |
| Ash, % | 1.41 ± 0.10^ab^ | 1.22 ± 0.03^b^ | 1.64 ± 0.14^a^ |
| Protein, % | 8.33 ± 0.12^b^ | 7.41 ± 0.13^c^ | 10.16 ± 0.16^a^ |
| Fat, % | 2.23 ± 0.12^b^ | 2.80 ± 0.17^a^ | 3.09 ± 0.01^a^ |
| Carbohydrates, % | 76.80 ± 0.24^a^ | 76.23 ± 0.22^b^ | 72.22 ±0.07^c^ |
| Fiber, % | 0.44 ± 0.105^a^ | 0.35 ± 0.12^a^ | 0.52 ± 0.17^a^ |
| Total polyphenols, mg GAE/Kg | 1288.11 ± 40.70^c^ | 2449.09 ± 97.47^b^ | 3265.92 ± 137.82^a^ |

Data are presented as mean ± standard deviation (n = 3). Values with different letters a, b and c in the same line are significantly different (p < 0.05).

***1.2.SM. Moisture evolution and appearance of rice flours during SAHT***

The moisture content of all rice flour samples increased after the steam-assisted heat treatment, reflecting the uptake of water from the steam-enriched oven environment. Despite the differences in inherent composition among brown, red, and black rice flours, all samples were conditioned to the same initial moisture content (12%), which minimized the initial variability and allowed a direct comparison of water absorption during treatment. The final moisture contents were estimated to be approximately 15.02 ± 0.15%, 14.87 ± 0.33%, and 14.61 ± 0.12% for brown, red, and black rice flours, respectively (not significant different). The slightly higher moisture retention observed in brown rice flour can be attributed to its higher starch content (76.8%) and lower fat content (2.23%) compared to black rice flour, which exhibited the lowest moisture gain. Starch is highly hydrophilic, providing abundant hydroxyl groups capable of forming hydrogen bonds with water, whereas fat is hydrophobic and can limit water absorption. The intermediate behavior of red rice flour aligns with its moderate starch and fat contents.

These findings are consistent with the principles of moisture sorption in cereal flours, where water uptake is determined by the balance between hydrophilic starch and protein fractions, the presence of lipids, and the availability of free water. Although black rice flour has a higher protein content (10.16%) than brown and red flours, its lower starch content and higher fat content likely limited its total water uptake. Additionally, the ventilated nature of the oven and the thin flour layer ensured uniform heat and moisture exposure, contributing to the relatively small differences (<0.5%) among the flours. Overall, the results indicate that SAHT increases flour moisture modestly, with compositional differences exerting only a minor influence when initial moisture is standardized. This highlights the importance of controlling initial hydration when assessing the impact of thermal treatments on flour physicochemical properties, as slight variations in water content can significantly affect subsequent starch gelatinization, pasting behavior, and enzymatic digestibility.

Photographic images of the rice flour samples were captured using a Canon 80D DSLR camera. Microscopic analysis was performed with an Optika-B290 microscope (Optika, Italy) featuring a 360° rotating head, 300° inclined eyepiece, adjustable interpupillary distance (48–75 mm), and a double-layer mechanical stage (150 × 139 mm) with 75 × 33 mm X–Y movement range and 0.1 mm accuracy. The system includes a 3.1 MP integrated camera and N-PLAN objectives (4×/0.10, 10×/0.25, 40×/0.65, and 100×/1.25), enabling detailed sample examination.

Brown rice flour

Red rice flour

Black rice flour

Figure 1.SM. Appearance of brown, red and black rice flour during exposure to steam-assisted heat treatment

The appearance of the rice flours during treatment can be seen in Figure 1.SM. Comparative analysis of the images indicates that SAHT induces progressive but variety-dependent visual changes in brown, red, and black rice flours. Brown rice flour exhibits the most pronounced and rapid color transition, changing from a light cream color to a uniform golden-brown within 30-50 minutes, suggesting extensive starch gelatinization and more evident non-enzymatic browning. Red rice flour shows a more gradual and less intense color change, with slight darkening and increased uniformity developing mainly by 40-50 minutes, likely moderated by the presence of native phenolic compounds that influence heat-induced reactions. Black rice flour displays the slowest and most complex transformation, shifting from a heterogeneous greyish-purple to a darker brownish tone as heating progresses, reflecting both thermal modification of starch and partial degradation or redistribution of anthocyanin pigments. Across all three flours, visible changes are most pronounced during the early to mid-stages of treatment (up to ~50–60 minutes), after which color stabilization suggests that major physicochemical transformations have largely been completed.

**1.3.SM. Description of the Moisture Environment During Steam-Assisted Heat Treatment**

To provide additional information regarding the moisture conditions during steam-assisted heat treatment, 250 mL of distilled water was placed inside the 65 L oven chamber before heating. This quantity corresponds to approximately 250 g of water, or 13.9 mol H₂O.

During treatment at 100 °C, the added water served as a continuous source of evaporation, generating a moisture-enriched atmosphere within the oven chamber. Because the oven operated as a ventilated system, water vapor was continuously exchanged with the surrounding environment. Consequently, the actual relative humidity inside the chamber was not measured and cannot be reliably determined from theoretical thermodynamic calculations.

Therefore, the purpose of the added water was not to establish a controlled saturated-steam atmosphere, but rather to maintain moisture availability throughout the thermal treatment. For this reason, the process is more appropriately described as a moist-heat treatment generated by continuous in situ evaporation of water in a ventilated oven.

The presence of moisture during treatment was confirmed experimentally by the increase in flour moisture content. All rice flours were initially standardized to 12% moisture and, after treatment, reached moisture contents of approximately 15.02 ± 0.15%, 14.87 ± 0.33%, and 14.61 ± 0.12% for brown, red, and black rice flours, respectively. These results demonstrate that the samples absorbed water during processing and confirm their exposure to a humid thermal environment.

Overall, the treatment conditions were defined by fixed operating parameters, including oven type and volume, water volume, temperature, treatment time, sample mass, and sample layer thickness, ensuring consistent processing conditions across all experimental runs.

***1.4.SM. FTIR spectra***

The iterative spectral deconvolution process for starch and protein secondary structures is illustrated in Figure 2.SM.

|  |  |
| --- | --- |
|  |  |
|  |  |
|  |  |

Evolution sequences of the iterative spectral deconvolution process for starch crystallinity

|  |  |
| --- | --- |
|  |  |
|  |  |
|  |  |

Evolution sequences of the iterative spectral deconvolution process for protein secondary structure

Figure 2.SM. Sequences from the evolution of the iterative spectral deconvolution process for monitoring starch crystallinity and protein secondary structure during steam-assisted heat treatment exposure

***1.5.SM. Image analysis***

Figure 3.SM. Histograms showing the area distribution of damaged regions identified on starch granules from brown rice flour (a), red rice flour (b), and black rice flour (c), before and after steam-assisted heat treatment.

***1.6.SM. Atomic force microscopy (AFM)***

| Not treated starch (Pintilii et al., 2025) | Steam-assisted treatment for 90 min |
| --- | --- |
| Starch isolated from brown rice flour | |
|   Ra (nm) = 414.1; Rq (nm) = 512.2 |   Ra (nm) = 91; Rq (nm) = 120 |
| Starch isolated from red rice flour | |
|   Ra (nm) > 1254; Rq (nm) > 1311 |   Ra (nm) = 552; Rq (nm) = 646 |
| Starch isolated from black rice flour | |
|   Ra (nm) = 307; Rq (nm) = 388 |   Ra (nm) = 482; Rq (nm) = 672 |

Figure 4.SM. 2D and 3D topographic atomic force microscopy (AFM) images of starch granules from brown rice flour, red rice flour, and black rice flour before and after exposure to steam-assisted heat treatment, with a scanned area of 10 x 10 μm².

***1.7. Starch digestion***

Table 2.SM. Changes in starch fraction composition of brown, red, and black rice flour during steam-assisted heat treatment: BRF0, BRF30, BRF60, BRF90 – starch isolated from brown rice flour subjected to steam-assisted treatment for 0, 30, 60 and 90 min; RRF0, RRF30, RRF60, RRF90 – starch isolated from red rice flour subjected to steam-assisted treatment for 0, 30, 60 and 90 min; BlRF0, BlRF30, BlRF60, BlRF90 – starch isolated from black rice flour subjected to steam-assisted treatment for 0, 30, 60 and 90 min.

| Starch type | Easily digested starch, % | Slowly digested starch, % | Resistant starch, % |
| --- | --- | --- | --- |
| BRF0 | 48.74 ± 1.74^αa^ | 27.44 ± 1.15^γc^ | 23.82 ± 0.74^βa^ |
| BRF30 | 34.22 ± 1.04^βb^ | 31.12 ± 0.94^βc^ | 34.66 ± 1.01^αa^ |
| BRF60 | 32.11 ± 1.34^βγa^ | 34.61 ± 1.10^αb^ | 33.28 ± 1.00^αa^ |
| BRF90 | 29.08 ± 1.36^γa^ | 36.87 ± 1.10^αb^ | 34.05 ± 0.87^αa^ |
|  |  |  |  |
| RRF0 | 45.23 ± 1.20^αb^ | 32.11 ± 0.82^γb^ | 22.66 ± 0.76^βa^ |
| RRF30 | 35.44 ± 1.07^βab^ | 34.54 ± 0.43^βb^ | 30.02 ± 0.77^αb^ |
| RRF60 | 34.22 ± 0.63^βab^ | 37.22 ± 1.17^αab^ | 28.56 ± 0.86^αb^ |
| RRF90 | 30.67 ± 0.68^γa^ | 39.09 ± 0.97^αab^ | 30.24 ± 0.61^αb^ |
|  |  |  |  |
| BlRF0 | 43.55 ± 0.49^αb^ | 36.42 ± 1.25^βa^ | 20.03 ± 0.53^γb^ |
| BlRF30 | 37.82 ± 0.94^βa^ | 38.71 ± 1.17^αβa^ | 23.47 ± 0.60 ^βc^ |
| BlRF60 | 35.06 ± 1.16^γa^ | 40.11 ± 1.56^αa^ | 24.83 ± 0.77 ^βc^ |
| BlRF90 | 31.02 ± 0.79^δa^ | 42.06 ± 1.58^αa^ | 26.92 ± 1.04^αc^ |

Data are presented as mean ± standard deviation (n = 3). Values with different letters a, b and c in the same column for different starch types but subjected to the same treatment time are significantly different (p < 0.05). Values with different letters α to γ in the same column for the same starch type but subjected to different exposure time are significantly different (p < 0.05).

***1.8.SM. Surface hydrophobicity of rice protein isolate***

Surface hydrophobicity of solid protein isolates was measured using the sessile drop method to assess wettability and surface properties of aggregated proteins after SAHT treatment. Unlike solution-based assays (e.g., ANS binding), this method evaluates water interaction with the solid surface, providing insight into surface roughness, porosity, and exposure of hydrophobic domains that influence texture, water absorption, and functional properties. Although less sensitive than solution-based methods, this approach provides valuable complementary information on the physical state and surface morphology of proteins after treatment.

Protein isolates were spread on a glass slide, and 5 μL of distilled water was applied (Zhao et al., 2021). Images were captured using a Canon EF 100mm f/2.8L Macro IS USM lens at 1× magnification and f/13. Contact angles on both sides of each drop were measured in ImageJ (Schneider et al., 2012) within 15 seconds to avoid water absorption. Contact angles were measured on both sides of each drop within 15 seconds to minimize water absorption by the sample. No additional image processing was applied.

| Not treated rice flour  (Pintiliii et al., 2025) | Protein isolate from rice flour subjected to steam-assisted heat treatment  for 60 min | Protein isolate from rice flour subjected to steam-assisted heat treatment  for 90 min |
| --- | --- | --- |
|  |  |  |
| Protein isolate derived from brown rice flour | | |
|  |  |  |
| 62.23^0^ ± 1.55^bα^ | 53.91 ± 2.56^cα^ | 67.12 ± 2.34^aα^ |
| Protein isolate derived from red rice flour | | |
|  |  |  |
| 47.29^0^ ± 0.89^aβ^ | 38.54 ± 3.11^cβ^ | 42.11 ± 2.87^bβ^ |
| Protein isolate derived from black rice flour | | |
|  |  |  |
| 39.13^0^ ± 0.61^aγ^ | 30.21 ± 1.77^cγ^ | 35.05 ± 2.12^bγ^ |

Figure 5.SM. Changes in the hydrophobicity of protein isolates from brown rice flour, red rice flour, and black rice flour during steam-assisted heat treatment.

Data are presented as mean ± standard deviation (n ≥ 10). Values with different letters a, b and c in the same line are significantly different (p ≤ 0.05). Values with different letters α, β and γ in the same column are significantly different (p ≤ 0.05).

The evolution of water contact angles for brown rice protein isolate under SAHT reflects the dynamic interplay between protein unfolding, thiol exposure, and oxidative cross-linking. The native protein exhibited a contact angle of 62.23°, indicative of a moderately hydrophobic surface. This is consistent with its structural characteristics: low free sulfhydryl content (0.41 mM/g), reflecting buried cysteine residues and limited exposure of polar groups to the aqueous environment. After 60 min of heating, the contact angle decreased to 53.91°, suggesting increased surface hydrophilicity. Mechanistically, this corresponds to partial protein unfolding and the exposure of previously buried polar residues, including thiol groups, as confirmed by the substantial rise in free sulfhydryl content (~73% increase). The decrease in contact angle implies enhanced water spreading, which would favor solubility and hydration, supporting functional applications such as gelation and emulsification. Prolonged heating to 90 min resulted in an increase in contact angle to 67.12°, exceeding the native value. This shift indicates a transition to a more hydrophobic surface, attributable to extensive thiol oxidation and disulfide bond formation, which stabilizes protein aggregates and buries previously exposed polar groups. The formation of a rigid, disulfide-cross-linked network reduces surface wettability, consistent with decreased solubility and altered gelation behavior observed in heat-treated proteins. Overall, the contact angle evolution demonstrates a two-stage mechanism: an initial hydrophilic phase driven by unfolding and thiol exposure, followed by a hydrophobic phase resulting from oxidative aggregation. These findings correlate closely with the measured thiol–disulfide balance and provide mechanistic insight into the functional consequences of thermal processing on brown rice protein surfaces.

The water contact angle of red rice protein isolate under SAHT exhibits a moderate decrease followed by a slight recovery, reflecting structural changes and thiol–disulfide dynamics. The native protein displayed a contact angle of 47.29°, lower than that of brown rice, indicating a more hydrophilic surface. This aligns with its structural properties: compact protein with limited cysteine exposure (free –SH = 0.36 mM/g) but potentially more surface-exposed polar residues compared to brown rice protein. After 60 min of heating, the contact angle decreased to 38.54°, reflecting increased surface hydrophilicity. This change is consistent with partial unfolding and exposure of buried polar residues, including thiol groups, which enhances water spreading. Functionally, this would improve solubility, hydration, and water-binding capacity. Prolonged heating to 90 min led to a slight increase in contact angle to 42.11°, indicating a minor reduction in surface hydrophilicity. Mechanistically, this corresponds to partial thiol oxidation and moderate disulfide bond formation, as the protein undergoes controlled aggregation. Importantly, free –SH levels remain elevated relative to the native state, suggesting that the surface retains significant polar character. This explains why the contact angle does not rise sharply, in contrast to brown rice protein. Overall, red rice protein isolate exhibits a more balanced response to heating compared to brown rice protein isolate: initial unfolding increases hydrophilicity, followed by moderate aggregation and oxidation that slightly reduces water wettability. The measured contact angle trend (47.29° → 38.54° → 42.11°) supports the notion of a controlled two-stage mechanism with retained surface polarity, highlighting the distinct thermal behavior and functional properties of red rice protein.

The water contact angle of black rice protein isolate under SAHT demonstrates consistently high hydrophilicity, reflecting structural flexibility and thiol exposure. The native protein exhibited a contact angle of 39.13°, the lowest among the three rice protein types, indicating a highly hydrophilic surface. This aligns with structural data showing moderate free –SH content (0.32 mM/g) and likely greater exposure of polar residues compared to brown and red rice proteins. After 60 min of heating, the contact angle decreased to 30.21°, reflecting enhanced hydrophilicity due to partial unfolding and significant exposure of buried thiol groups, as well as other polar residues. This structural change favors water spreading, solubility, and hydration, highlighting black rice protein isolate high functional potential in aqueous systems. Prolonged heating to 90 min resulted in a slight increase in contact angle to 35.05°, indicating a minor reduction in surface wettability. Mechanistically, this corresponds to moderate oxidative cross-linking and partial disulfide formation, but the overall thiol exposure remains high. Consequently, the protein surface retains considerable polar character, preventing a sharp increase in contact angle, unlike brown rice protein. Overall, black rice protein isolate exhibits a gradual two-stage behavior, with an initial hydrophilic phase driven by unfolding and thiol exposure, followed by moderate aggregation that slightly reduces wettability. The measured contact angle trend (39.13° → 30.21° → 35.05°) supports its high surface polarity and sustained water affinity, which distinguishes black rice protein from brown and red rice proteins in thermal stability and functional performance.

The evolution of water contact angle in rice protein isolates under SAHT is closely linked to changes in thiol–disulfide chemistry and secondary structure. In brown rice protein, the contact angle decreased from 62.23° to 53.91° after 60 min due to partial unfolding and exposure of free sulfhydryl groups, enhancing surface hydrophilicity. Prolonged heating to 90 min induced extensive disulfide formation and aggregation, burying polar residues and increasing the contact angle to 67.12°. Red rice protein exhibited a similar initial decrease (47.29° → 38.54°) but only a slight increase to 42.11° after 90 min, reflecting moderate disulfide cross-linking and retention of free –SH, which preserved surface polarity. Black rice protein displayed the lowest contact angles overall (39.13° → 30.21° → 35.05°), consistent with continuous thiol exposure, limited disulfide formation, and sustained unfolding, maintaining a highly hydrophilic surface. These trends indicate a strong correlation between free –SH content, disulfide bond formation, secondary structure changes, and surface wettability, demonstrating that proteins with higher free –SH and less aggregation exhibit lower contact angles, whereas extensive oxidation and aggregation lead to hydrophobic surfaces. This mechanistic relationship provides insight into the functional behavior of rice proteins under thermal processing, including solubility, hydration, and gelation potential.

***1.8.SM. PCA analysis***

Table 3.SM. The variability of individual and cumulative data for the main components associated to starch relative crystalline degree and secondary structure of protein before and after steam-assisted heat treatment

| PC | eigenvalue | % total variance | cumulative eigenvalue | cumulative, % |
| --- | --- | --- | --- | --- |
| Before treatment (Pintilii et al., 2025) | | | | |
| 1 | 106.16 | 94.67 | 106.16 | 94.67 |
| 2 | 5.62 | 5.01 | 111.78 | 99.68 |
| 3 | 0.26 | 0.24 | 112.05 | 99.91 |
| 4 | 0.10 | 0.09 | 112.14 | 100.00 |
| After steam-assisted heat treatment | | | | |
| 1 | 174.64 | 48.66 | 174.64 | 48.66 |
| 2 | 148.77 | 41.45 | 323.40 | 90.12 |
| 3 | 31.02 | 8.64 | 354.42 | 98.76 |
| 4 | 3.27 | 0.91 | 357.69 | 99.67 |
| 5 | 1.18 | 0.33 | 358.87 | 100.00 |

**References**

Pintilii, C., Mihaly Cozmuta, L., Nicula, C., Peter, A., & Mihaly Cozmuta, A. (2025). The effect of microwave treatment on polyphenols, flavonoids, antioxidant capacity of colored rice. *Paper presented at the Multidisciplinary Conference on Sustainable Development Section: Food Chemistry, Engineering & Technology.* University of Life Sciences “King Mihai I” from Timişoara, Timisoara.

Schneider, C. A., Rasband, W. S., & Eliceiri, K. W. (2012). *ImageJ* (Version 1.47) [Computer software]. U.S. National Institutes of Health. <https://imagej.nih.gov/ij/>.

Wang, H., Yang, Q., Gao, L., Gong, X., Qu, Y., & Feng, B. (2020). Functional and physicochemical properties of flours and starches from different tuber crops. *International Journal of Biological Macromolecules, 148*, 324–332. https://doi.org/10.1016/j.ijbiomac.2020.01.146.

Zhao, Q., Lin, J., Wang, C., Yousaf, L., Xue, Y., & Shen, Q. (2021). Protein structural properties and proteomic analysis of rice during storage at different temperatures. *Food Chemistry,* *361*, Article 130028. https://doi.org/10.1016/j.foodchem.2021.130028.
